# Cooperative outer-surface recognition extends host–guest chemistry for high-capacity molecular separation beyond cavity inclusion

**DOI:** 10.1038/s41467-026-78531-8

**Published:** 2026-10-08

**Authors:** Yafang Zhang, Tao Hou, Lixia Chen, Zhu Tao, Xi-Sheng Wang, Qing Li

**Affiliations:** 1 School of Chemistry and Chemical Engineering, State Key Laboratory of Green Pesticide, Guizhou University, Guiyang, China; 2 Hefei National Research Center for Physical Sciences at the Microscale and Department of Chemistry, University of Science and Technology of China, Hefei, China

**Keywords:** Molecular capsules, Organic molecules in materials science, Organic molecules in materials science

## Abstract

The separation of azeotropic and structurally similar mixtures remains a fundamental challenge for conventional porous materials. Supramolecular host–guest chemistry provides exceptional recognition precision but is inherently limited by discrete cavity-confined binding. Here, we establish a strategy that extends host–guest recognition beyond cavity inclusion by activating cooperative interactions at the outer surfaces of macrocyclic hosts. Using cucurbit[7]uril (Q[7]) as a model system, we demonstrate that exposed outer surfaces can serve as amplifying recognition interfaces, where initial guest binding triggers subsequent molecular recruitment through cooperative aromatic interactions. Immobilization of Q[7] on magnetic Fe₃O₄ nanoparticles suppresses aggregation and enables synergistic cavity- and outer-surface-mediated recognition. The resulting material achieves highly selective and efficient separation of benzene (Ben) from *n*-heptane (*n*-Hep) with enhanced adsorption capacity and recyclability. This work suggests outer-surface-mediated cooperative recognition as a general paradigm for designing supramolecular separation materials beyond cavity-centric host–guest chemistry.

## Abstract (web summary)

The separation of azeotropic and structurally similar mixtures remains challenging for conventional porous materials. Here the authors introduce a conceptual extension of classical host–guest chemistry that moves beyond exclusive cavity-based recognition to exploit cooperative interactions at the outer surface of macrocyclic hosts.

## Introduction

The separation of azeotropic and structurally similar mixtures remains a persistent challenge in chemical engineering and industrial chemistry^1–3^. Conventional separation materials, including zeolites^4–7^, molecular sieves^8,9^, metal–organic frameworks (MOFs)^10–13^, and covalent organic frameworks (COFs)^14–17^, primarily rely on size exclusion, shape selectivity, or differential adsorption energies within rigid or semi-rigid pores. Despite substantial progress, their performance often deteriorates when target components exhibit nearly identical kinetic diameters, boiling points, and physicochemical properties, making it fundamentally difficult to achieve both high selectivity and high adsorption capacity under energy-efficient conditions^18^.

Supramolecular recognition provides a conceptually distinct strategy by shifting separation from bulk material properties to molecular-level interactions^19–21^. Through non-covalent forces such as hydrogen bonding, π···π interactions, and electrostatic effects, supramolecular systems can discriminate between molecules with subtle structural or electronic differences^22,23^. Within this framework, host–guest chemistry constitutes a foundational paradigm, in which macrocyclic hosts selectively encapsulate guest molecules within well-defined cavities^24–26^. While this cavity-centric recognition enables high selectivity, the inherently discrete nature of inclusion—typically accommodating only one or two guest molecules per host—severely limits adsorption capacity and hinders practical large-scale applications ^27–29^.

**Fig. 1:**
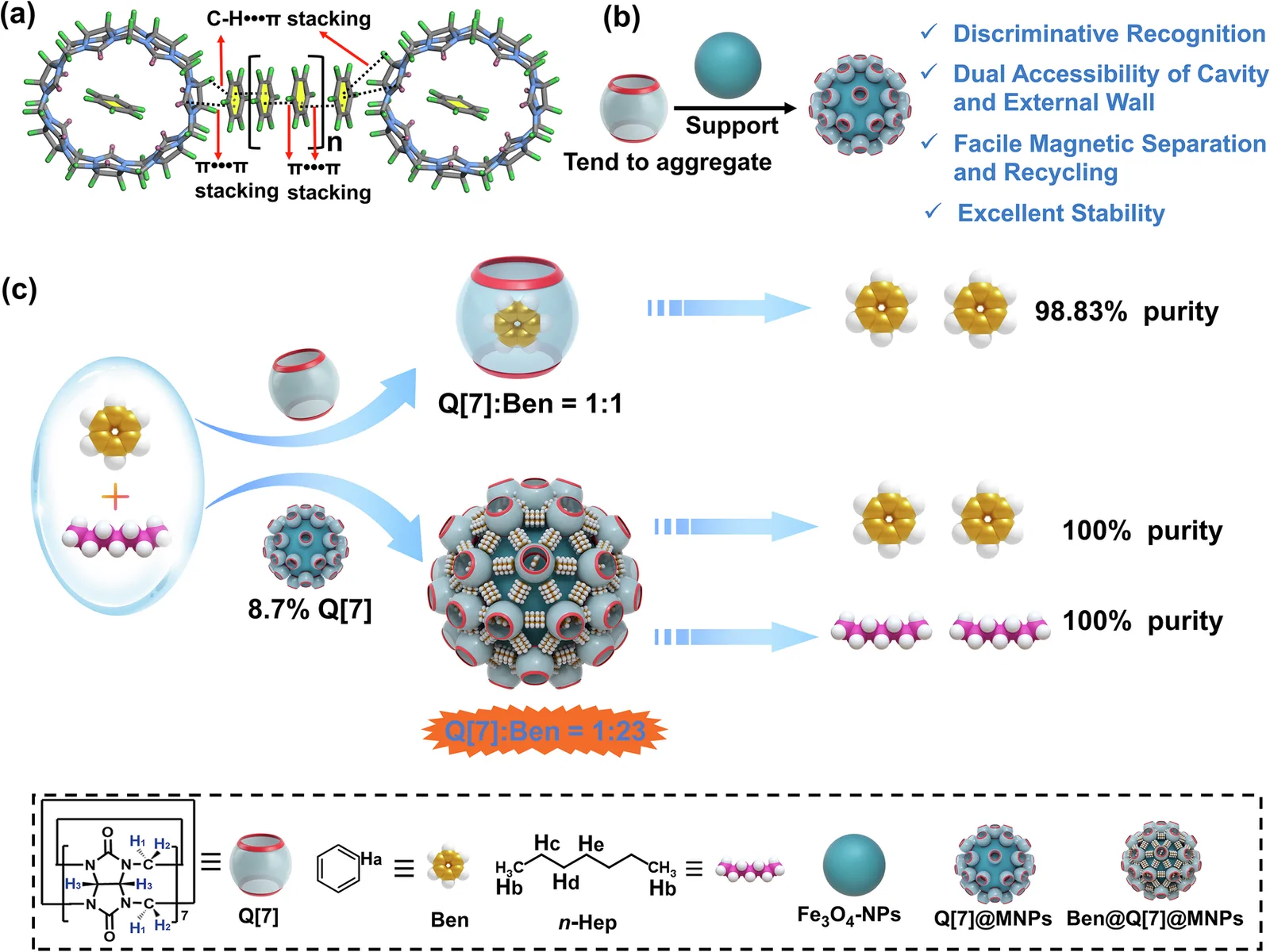
Conceptual design of outer-surface-mediated cooperative recognition and Q[7]@MNPs-based separation strategy. **a** Outer-Surface Interactions between Q[7] and Aromatic Molecules. **b** Synthetic route to Q[7]@MNPs. **c** The schematic illustration of the separation process for Ben and *n*-Hep using Q[7] and Q[7]@MNPs.

Here we introduce a conceptual extension of host–guest chemistry that moves beyond exclusive reliance on cavity inclusion. Macrocyclic hosts intrinsically possess two spatially distinct recognition domains—an internal cavity and an external surface—yet the latter has largely been regarded as structurally passive^30^. We propose that interactions occurring at the macrocyclic exterior represent a legitimate and underutilized mode of host–guest recognition that can operate cooperatively (Fig. 1a). In this model, an initial binding event at an exposed macrocyclic surface serves as a molecular anchor that recruits additional guest molecules through intermolecular interactions until a cooperatively stabilized interfacial assembly is formed; for aromatic compounds, successive π···π stacking gives rise to a cascade adsorption process, translating subtle molecular recognition into macroscopic adsorption enhancement and effectively decoupling selectivity from capacity.

We validate this concept using cucurbiturils^31–34^ as a representative macrocyclic host system. Although cucurbituril chemistry has been dominated by carbonyl-lined cavity inclusion^35–38^, their rigid outer walls are capable of engaging in aromatic interactions^39^. By immobilizing Q[7] onto magnetic Fe₃O₄ nanoparticles, aggregation is suppressed and both cavity-based and outer-surface-based recognition sites are rendered accessible (Fig. 1b). The resulting Q[7]@MNPs material enables the highly selective separation of Ben from *n*-Hep, a prototypical aromatic/aliphatic azeotropic system^40^, exhibiting dramatically enhanced adsorption capacity, complete selectivity, and excellent recyclability (Fig. 1c). More broadly, this work establishes outer-surface-mediated recognition as a general extension of classical host–guest chemistry and provides a design principle for transforming macrocyclic hosts into high-capacity, selective separation materials.

## Results

### Characterization of the adsorbent

**Fig. 2:**
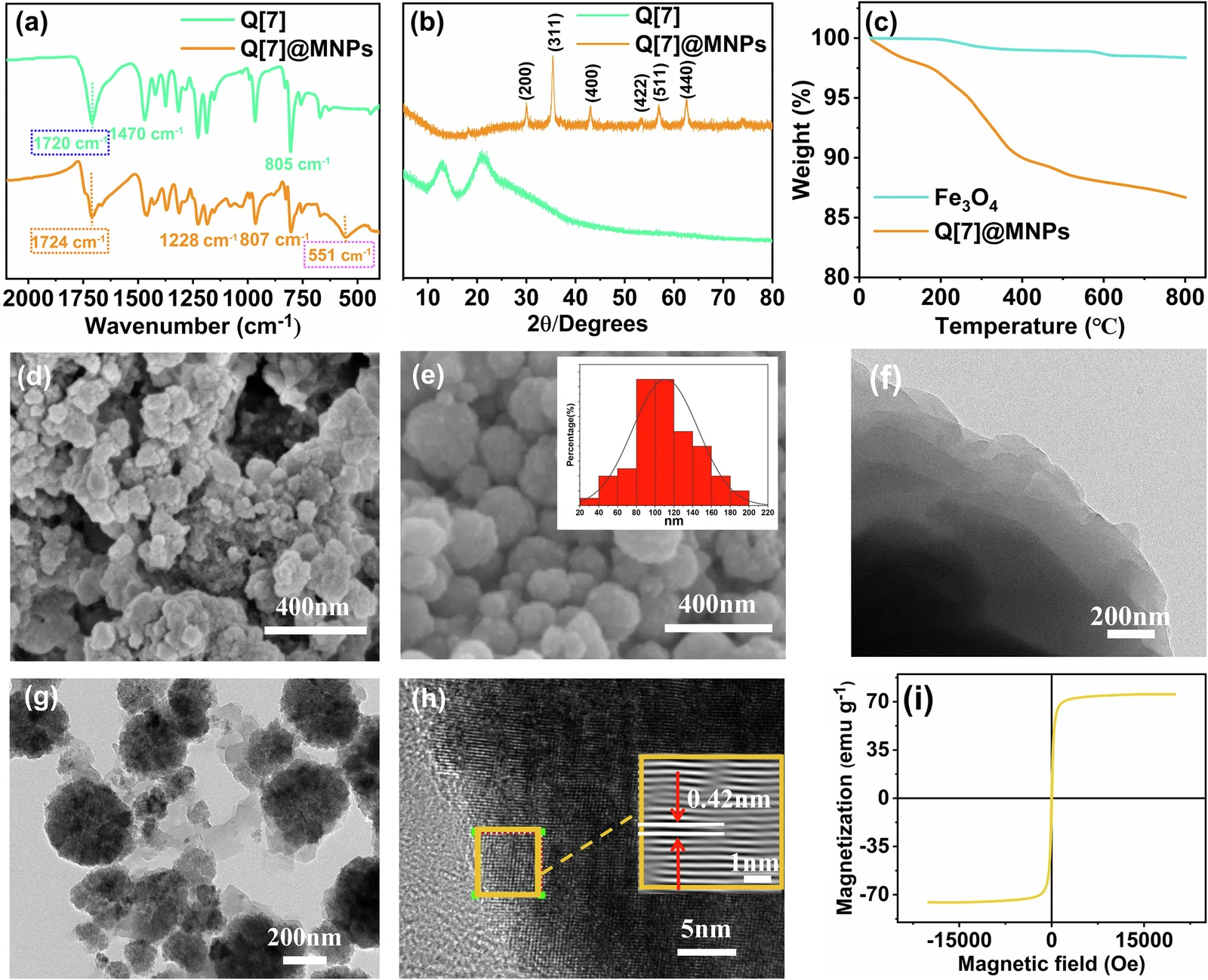
Structural characterization of Q[7]@MNPs. **a** FT-IR spectra of Q[7] and Q[7]@MNPs. **b** XRD patterns of Q[7] and Q[7]@MNPs. **c** TG curves of Fe_3_O_4_ and Q[7]@MNPs. **d** Representative SEM image of Q[7]. **e** Representative SEM image of Q[7]@MNPs. The inset shows the particle size distribution histogram (based on 200 nanoparticles). **f** Representative TEM image of Q[7]. **g** Representative TEM image of Q[7]@MNPs. **h** High-resolution TEM image of Q[7]@MNPs. **i** Magnetization hysteresis loop of Q[7]@MNPs.

The successful fabrication of Q[7]@MNPs was first confirmed by Fourier-transform infrared (FT-IR) spectroscopy (Fig. 2a). A characteristic absorption band at 551 cm^−1^ is assigned to the Fe–O stretching vibration of iron oxide nanoparticles. The strong band at ca. 1720 cm^−1^ corresponds to the C═O stretching vibration of the glycoluril units in Q[7], while the absorption at 1228 cm^−1^ is attributed to C–N stretching. Notably, Q[7]@MNPs display a pronounced C═O stretching band at 1724 cm^−1^, providing clear evidence for the presence of Q[7] on the nanoparticle surface.

Powder X-ray diffraction (XRD) patterns of Q[7]@MNPs (Fig. 2b) exhibit broad diffraction peaks characteristic of nanocrystalline magnetite, with all reflections matching well with Fe₃O₄ (JCPDS No. 19-0629). Thermogravimetric analysis (TGA) further supports successful hybridization. Compared with bare Fe₃O₄, which shows a minor weight loss of ca. 2%, Q[7]@MNPs display a pronounced mass loss of ~18.2 wt% between 50 and 800 °C (Fig. 2c), attributable to the decomposition of organic components. Combined elemental analysis and TGA results indicate a Q[7] loading of 8.70 wt% on the magnetic nanoparticles (Table S1).

Scanning electron microscopy (SEM) and transmission electron microscopy (TEM) were employed to examine the morphology and dispersion of the materials. Pristine Q[7] exhibits a particle morphology (Fig. 2d), whereas Q[7]@MNPs consist of nearly spherical nanoparticles with uniform size distribution (Fig. 2e). Statistical analysis based on more than 200 particles reveals diameters predominantly in the range of 80–120 nm. Energy-dispersive X-ray spectroscopy (EDS) elemental mapping (Fig. S1) shows that the characteristic C and N signals originating from Q[7] are uniformly distributed over the MNP surface without noticeable localized enrichment, indicating homogeneous immobilization of Q[7] on the nanoparticles and the absence of large-scale aggregation. TEM images further confirm the distinct morphological transformation upon immobilization (Fig. 2f, g), with Q[7]@MNPs showing significantly improved dispersion relative to bulk Q[7]. This enhancement is attributed to the anchoring of Q[7] carbonyl groups on the nanoparticle surface, which effectively suppresses aggregation. High-resolution TEM reveals clear lattice fringes with an interplanar spacing of 0.42 nm (Fig. 2h), consistent with magnetite crystallography.

Magnetic properties were evaluated using a vibrating sample magnetometer (VSM). Q[7]@MNPs exhibit a saturation magnetization of 75.36 emu g^−1^ with negligible coercivity and remanence (Fig. 2i), indicating superparamagnetic behavior (Supplementary Video 1 and Fig. S2). The material responds rapidly to an external magnetic field, enabling efficient magnetic separation during adsorption experiments and highlighting its practical operability.

To evaluate the structural contribution to adsorption, N₂ adsorption–desorption measurements were performed (Fig. S3 and Table S2). Based on the adsorption isotherm collected at 77 K, the Brunauer–Emmett–Teller (BET) specific surface area of Q[7]@MNPs was determined to be 16.44 m² g⁻¹, with a narrow pore size distribution centered at ~3.6 nm. These values indicate that Q[7]@MNPs does not possess the high specific surface area or well-defined microporous architecture characteristic of conventional porous adsorbents. Therefore, the exceptional Ben adsorption performance is unlikely to arise primarily from surface-area or pore-confinement effects, suggesting instead that molecular recognition by immobilized Q[7] plays the dominant role.

### Solid–liquid phase separation experiment

**Fig. 3:**
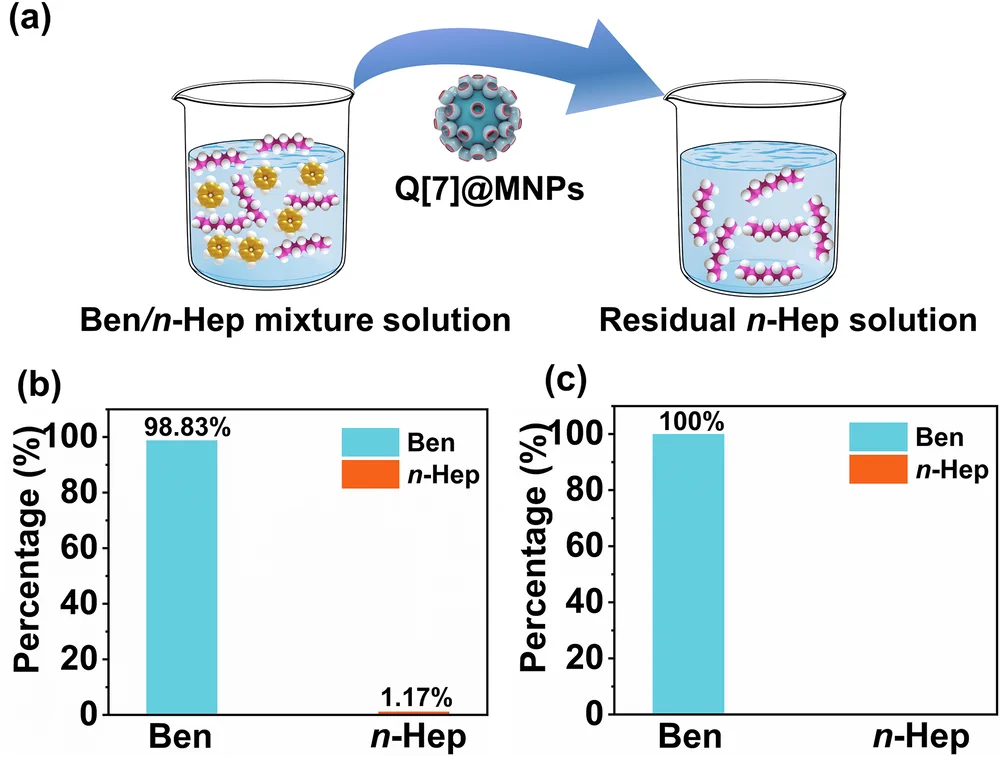
Selective separation of Ben from *n*-Hep using Q[7]@MNPs. **a** Schematic illustration of the separation of a Ben/*n*-Hep mixture (v_*n*-Hep_:v_Ben_ = 1:1) using Q[7]@MNPs. The relative adsorption of Ben and *n*-Hep by Q[7] **b** and Q[7]@MNPs **c** was quantified by GC.

The separation performance of Q[7]@MNPs was evaluated using a Ben/*n*-Hep mixture with a volume ratio of 1:1 (Fig. 3a). Quantitative analysis was performed by gas chromatography (GC). Control experiments confirm that bare Fe₃O₄ nanoparticles show negligible adsorption toward either component (Fig. S4). Pristine Q[7] exhibits preferential adsorption of Ben, affording a selectivity of 98.83% (Figs. 3b and S5–S7). The equilibrium adsorption stoichiometry is ~1:1 (Fig. S8), consistent with cavity-based aromatic recognition. Strikingly, Q[7]@MNPs display complete selectivity for Ben, reaching 100% under identical conditions (Figs. 3c and S9). This pronounced enhancement highlights the critical role of nanoparticle immobilization in activating additional recognition pathways beyond simple cavity inclusion.

### Adsorption kinetics and isotherm studies in the adsorption of Ben

**Fig. 4:**
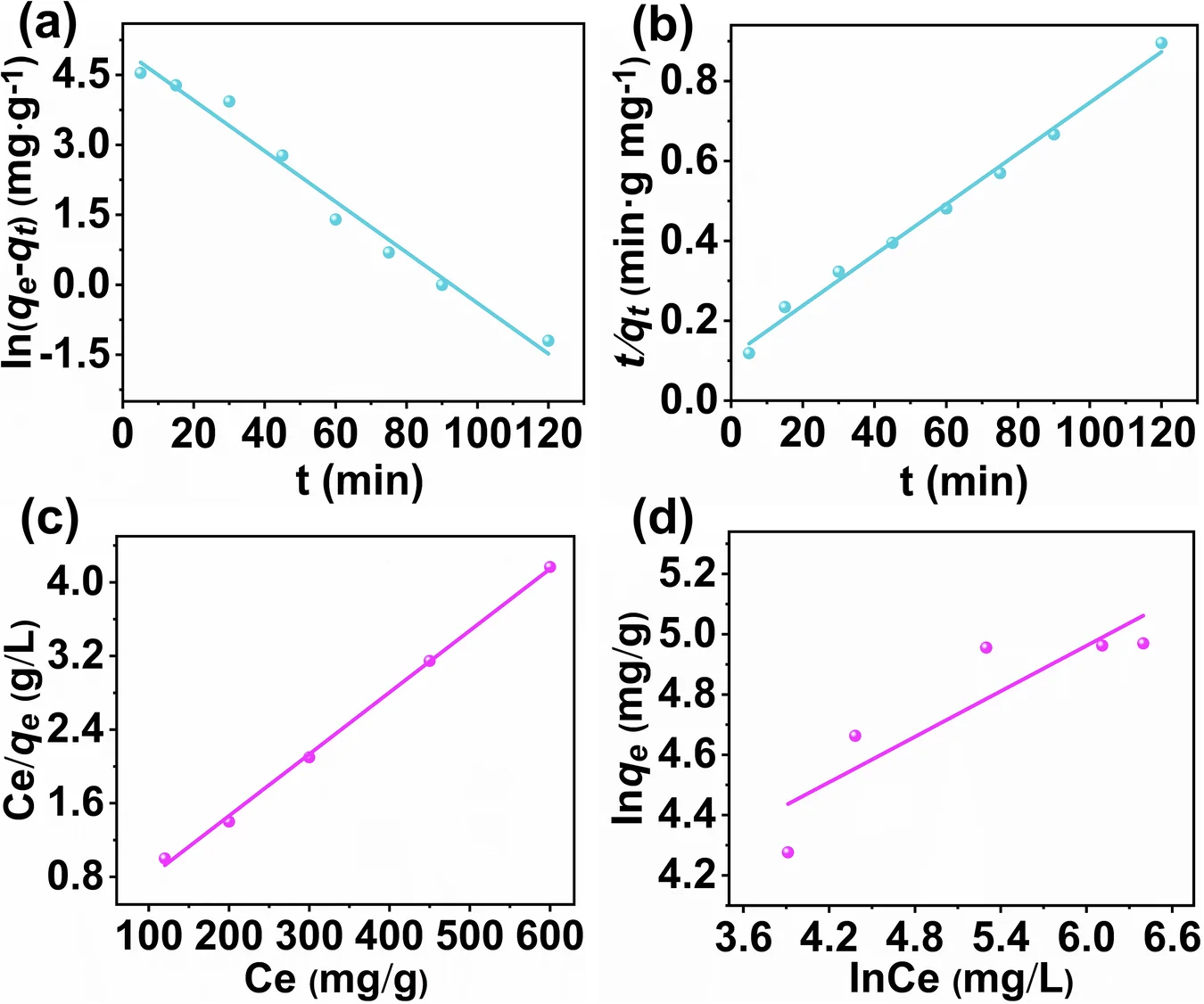
Adsorption kinetics and equilibrium analysis of Ben adsorption on Q[7]@MNPs. **a** Pseudo-first-order adsorption kinetics; **b** Pseudo-second-order adsorption kinetics; **c** Langmuir adsorption isotherm; **d** Freundlich adsorption isotherm.

To gain insight into the adsorption mechanism, kinetic and isotherm studies were conducted at room temperature. Time-dependent adsorption data for Ben on Q[7]@MNPs were fitted using pseudo-first-order and pseudo-second-order kinetic models (Fig. 4a,b and Table S3). The pseudo-second-order model provides a superior fit (*R*² = 0.99) compared with the pseudo-first-order model (*R*² = 0.97), indicating that the adsorption rate is governed by the availability of active sites and Ben concentration. Equilibrium is reached within ~45 min, with a maximum adsorption capacity of 136.17 mg g^−1^ (Fig. S10).

Adsorption isotherms were obtained by varying the initial Ben concentration and analyzed using Langmuir and Freundlich models (Fig. 4c, d and Table S4). The Langmuir model affords a higher correlation coefficient (*R*² = 0.99) than the Freundlich model (*R*² = 0.97), with a calculated maximum adsorption capacity (*q*_m_ = 149.03 mg g^−1^) in close agreement with experimental data. These results indicate monolayer adsorption on energetically uniform sites, consistent with a chemisorption-dominated process.

### Adsorption mechanism

Based on the Q[7] loading (8.70 wt%), the theoretical Ben uptake arising solely from cavity inclusion is estimated to be ~5.84 mg g^−1^. In sharp contrast, the experimentally observed saturation capacity reaches 136.17 mg g^−1^, representing an enhancement of more than two orders of magnitude. This discrepancy clearly indicates the presence of additional adsorption pathways beyond classical host–guest inclusion.

**Fig. 5:**
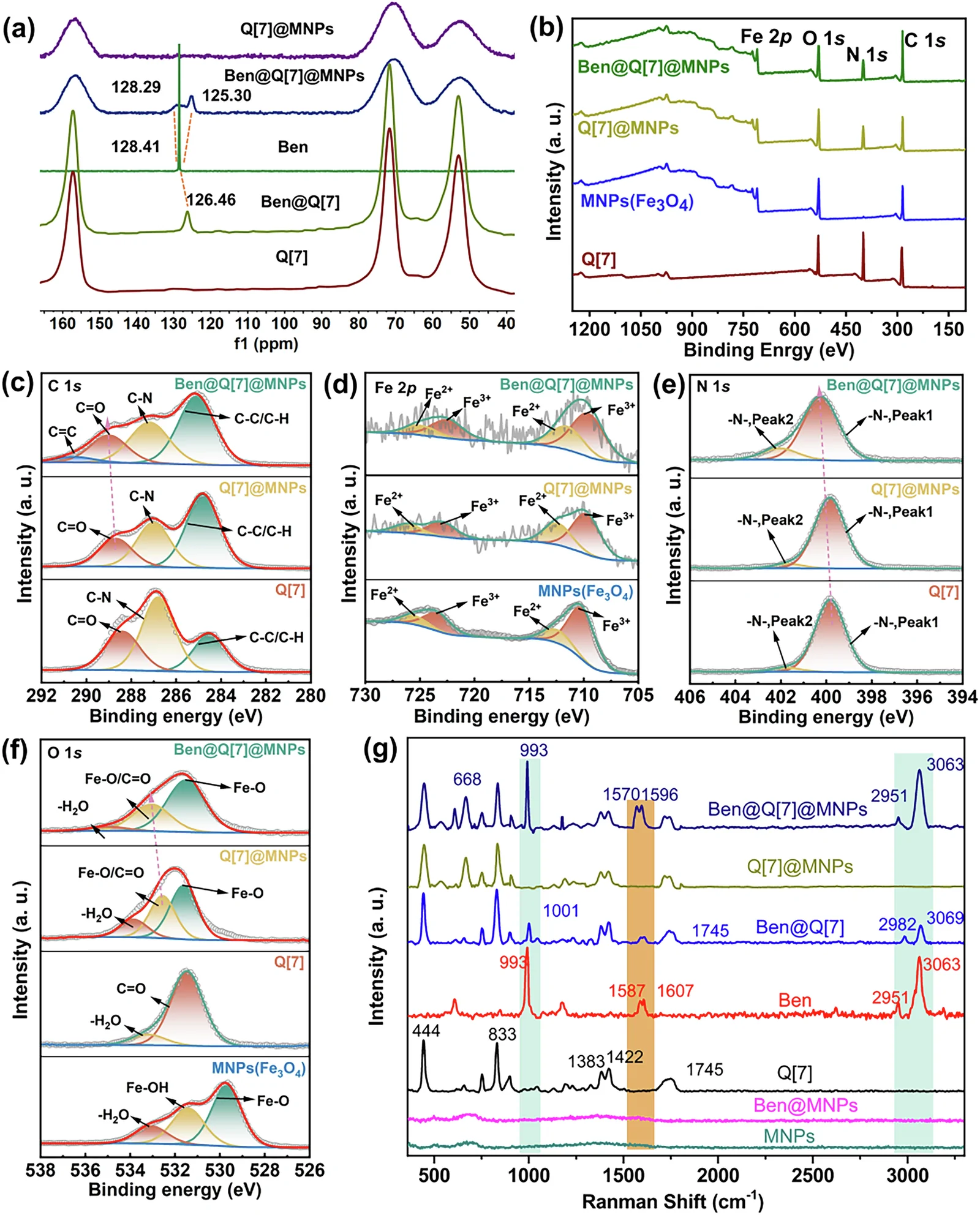
Experimental evidence for cooperative Ben recognition on Q[7]@MNPs. **a** Solid-state ¹³C CP/MAS NMR spectra of Q[7], Ben@Q[7], Ben, Ben@Q[7]@MNPs, and Q[7]@MNPs. **b** XPS survey spectra of Q[7], Fe₃O₄ MNPs, Q[7]@MNPs, and Ben@Q[7]@MNPs. **c**–**f** High-resolution XPS spectra of C 1 *s* (**c**), Fe 2*p* (**d**), N 1 *s* (**e**), and O 1 *s* (**f**) regions of the indicated samples. **g** Raman spectra of Fe₃O₄ MNPs, Ben@MNPs, Q[7], Ben, Ben@Q[7], Q[7]@MNPs, and Ben@Q[7]@MNPs.

To determine whether adsorption pathways beyond cavity inclusion exist, solid-state ¹³C CP/MAS NMR spectra were collected before and after Ben adsorption (Fig. 5a). Whereas pure Ben exhibited a single resonance at *δ* = 128.54 ppm, Ben@Q[7]@MNPs displayed two distinct aromatic carbon resonances at *δ* = 127.30 and 128.34 ppm. These two resonances indicate that Ben molecules experience two different chemical environments within the composite. Combined with the well-established host–guest behavior of cucurbiturils^41–43^ and the spectroscopic evidence presented below, these resonances are assigned to cavity-included Ben and Ben adsorbed on the exterior surface of Q[7], respectively. Therefore, the solid-state NMR results provide direct structural evidence for the coexistence of cavity inclusion and outer-surface adsorption.

The adsorption site was further investigated by X-ray photoelectron spectroscopy (Fig. 5b–f). Compared with Q[7]@MNPs, Ben@Q[7]@MNPs exhibited a pronounced aromatic C = C component in the C 1 *s* spectra (Fig. 5c) together with a substantial increase in overall carbon intensity, indicating significant enrichment of aromatic species on the material surface. In contrast, the Fe content and the Fe 2*p* spectra (Fig. 5d) remained essentially unchanged before and after adsorption, excluding Fe₃O₄ as the primary adsorption site. Considering that each Q[7] cavity accommodates only one Ben molecule through host–guest inclusion, the dramatic increase in aromatic carbon is more consistent with multisite adsorption on the exposed outer surface than with cavity inclusion alone. Moreover, both the N 1 *s* and O 1 *s* spectra (Fig. 5e, f) exhibited systematic shifts toward lower binding energies after Ben adsorption, indicating redistribution of the electronic environment throughout the Q[7] framework^39,44^. Such global electronic perturbation would be difficult to account for solely by localized cavity inclusion^45^ but is readily explained by extensive interactions between Ben molecules and the exposed exterior of immobilized Q[7]. These observations therefore provide strong spectroscopic evidence that the outer surface serves as an additional adsorption interface.

Raman spectroscopy further elucidated the intermolecular interactions responsible for the enhanced adsorption capacity (Figs. 5g and S11 and Tables S5 and S6). Compared with free Ben, the characteristic ring-breathing and symmetric C–H stretching vibrations of Ben@Q[7] shifted from 993 to 1001 cm⁻¹ and from 3063 to 3069 cm⁻¹, respectively, confirming the confined environment associated with cavity inclusion^46,47^. In comparison with Ben@Q[7], the Raman intensity of Ben became markedly stronger after immobilization on Q[7]@MNPs, consistent with adsorption of substantially more Ben molecules than permitted by the cavity alone. Meanwhile, the characteristic vibrations of Q[7] remained essentially unchanged except for slight peak shifts and line broadening, indicating that adsorption proceeds through non-covalent interactions without disrupting the macrocyclic framework^48,49^. More importantly, the aromatic C–C stretching mode (*ν*₈) and its associated Fermi resonance underwent pronounced red shifts from 1607 to 1596 cm⁻¹ and from 1587 to 1570 cm⁻¹, respectively. Such simultaneous red shifts are well-recognized Raman signatures of intermolecular π–π stacking^50^. Therefore, Raman spectroscopy reveals vibrational changes consistent with aromatic stacking interactions among adsorbed Ben molecules.

**Fig. 6:**
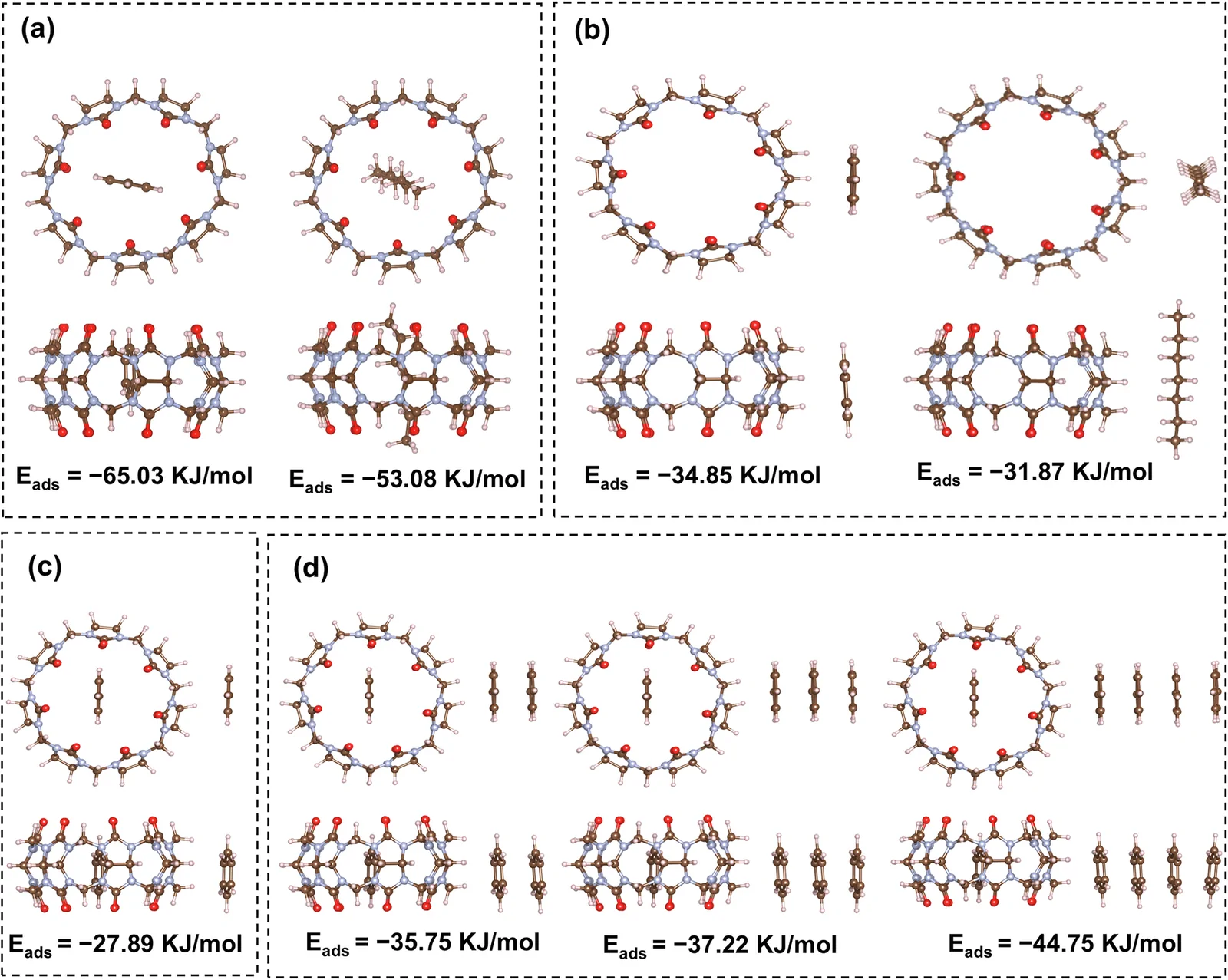
Theoretical insights into cooperative recognition modes. DFT calculations of adsorption mechanisms in the Q[7] system. **a** Cavity inclusion of Ben and *n*-Hep in Q[7]. **b** Outer-surface adsorption of Ben and *n*-Hep on Q[7]. **c** Dual-site adsorption involving simultaneous cavity and surface binding of Ben. **d** Cooperative π–π stacking of multiple Ben molecules (*n* = 2–4) on the Q[7] surface, showing progressively enhanced stabilization.

Density functional theory calculations are fully consistent with the experimental observations. Ben binds more strongly inside the Q[7] cavity than *n*-Hep, with calculated binding energies of −65.03 and −53.08 kJ mol⁻¹ (Fig. 6a), respectively, explaining the experimentally observed selectivity. Beyond cavity inclusion, adsorption of a single Ben molecule on the outer surface of Q[7] remains thermodynamically favorable (E_ads_ = −34.85 kJ mol⁻¹), whereas *n*-Hep interacts more weakly (−31.87 kJ mol⁻¹) (Fig. 6b), indicating that the exposed outer surface also preferentially recognizes aromatic guests. A dual-site adsorption model containing one cavity-included Ben molecule together with a second Ben adsorbed on the exterior remains energetically stable (E_ads_ = −27.89 kJ mol⁻¹) (Fig. 6c), demonstrating that the two adsorption modes can coexist. Furthermore, calculations on two-, three-, and four-molecule Ben assemblies adsorbed on the outer surface yielded progressively more favorable stabilization energies (−35.75, −37.22, and −44.75 kJ mol⁻¹, respectively) (Fig. 6d), demonstrating that intermolecular aromatic stacking provides an additional thermodynamic driving force for cooperative adsorption.

Collectively, the experimental and theoretical results support a hierarchical cooperative adsorption mechanism (Fig. 1a). Immobilization of Q[7] on Fe₃O₄ effectively suppresses macrocycle aggregation and exposes both the cavity and the positively polarized outer surface. Ben is preferentially captured through cavity inclusion while simultaneously interacting with the exposed outer surface through weak non-covalent interactions. The initially adsorbed Ben molecules subsequently recruit additional Ben molecules through intermolecular π–π stacking, generating a cascade adsorption process that dramatically amplifies adsorption capacity. In contrast, the non-aromatic *n*-Hep cannot participate in these cooperative interactions, thereby accounting for the outstanding Ben selectivity of Q[7]@MNPs.

### Cycling performance

**Fig. 7:**
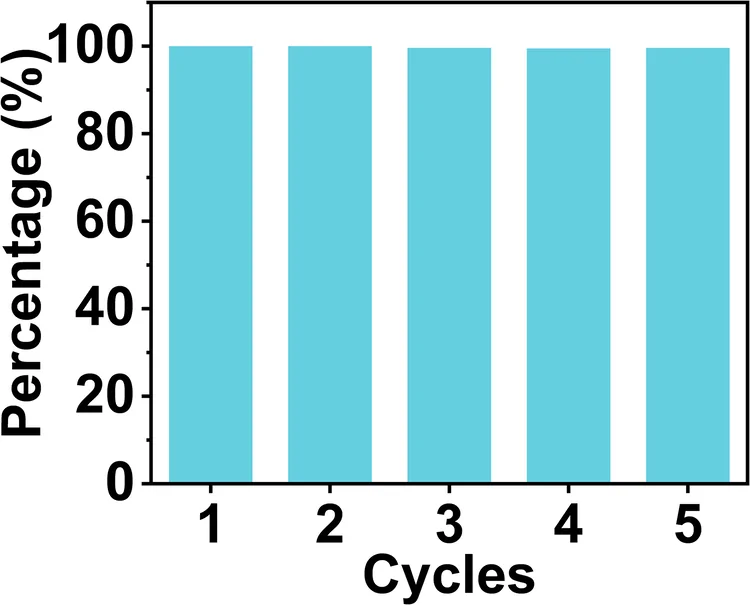
Recyclability of Q[7]@MNPs for Ben separation. Percentage of Ben separated by Q[7]@MNPs within five recycles.

Recyclability is a critical parameter for practical separation materials. After adsorption, Q[7]@MNPs were regenerated by heating at 120 °C under vacuum to remove adsorbed Ben. The regenerated material was reused for five consecutive separation cycles under identical conditions (Figs. S12–S16). GC analysis confirms that Ben selectivity remains above 99% throughout all cycles (Fig. 7), demonstrating excellent stability and recyclability.

### Purification of *n*-Hep

Given its high selectivity toward benzene (Ben), the capability of Q[7]@MNPs for the removal of trace Ben from *n*-heptane (*n*-Hep) was further assessed. Liquid–solid adsorption experiments were conducted using Ben/*n*-Hep mixtures (v_*n*-Hep_:v_Ben_ = 98:2) to simulate practical purification conditions. Upon treatment with Q[7]@MNPs for 1 h, the purity of *n*-Hep increased from 97.51% to 99.23%, accompanied by a decrease in the Ben content from 2.49 to 0.77% (Figs. S17–18). The result highlights the potential of Q[7]@MNPs for deep purification of aliphatic hydrocarbons in liquid phases.

## Discussion

In conclusion, we demonstrate that the outer wall of Q[7] can be transformed from a structurally passive boundary into an active and cooperatively amplifiable recognition interface for molecular separation. By suppressing macrocycle aggregation through immobilization on magnetic Fe₃O₄ nanoparticles, both cavity-based inclusion and outer-surface-mediated aromatic interactions are simultaneously activated, enabling a cascade adsorption mechanism that decouples molecular selectivity from adsorption capacity. As a result, the Q[7]@MNPs material achieves complete selectivity, markedly enhanced uptake, and excellent recyclability in the challenging separation of the Ben/*n*-Hep azeotrope. This work suggests outer-surface-mediated recognition as an extension of classical host–guest chemistry and identifies aggregation control combined with cooperative intermolecular interactions as a versatile design principle for converting discrete supramolecular hosts into high-capacity, solid-state separation materials, thereby bridging supramolecular recognition and materials engineering and opening new avenues for separation problems beyond the reach of conventional porous adsorbents and cavity-centric supramolecular strategies.

## Methods

### Materials

FeCl₃·6H₂O (99%) was purchased from Shanghai McLean Biochemical Technology Co., Ltd. Ethylene glycol (C₂H₆O₂), sodium acetate (CH₃COONa, 99.7%), ethanol (CH₃CH₂OH, ≥99.7%), and sodium citrate (C₆H₇NaO₇, 98%) were purchased from Shanghai Aladdin Biochemical Technology Co., Ltd. All chemicals were used without further purification. Q[7] was prepared in our laboratory. Ultra-pure deionized water was used in all experiments.

### Preparation of Q[7]@MNPs materials

Q[7]@MNPs were synthesized according to a previously reported solvothermal procedure with minor modifications^51^. FeCl₃·6H₂O (2.50 g, 9.2 mmol) and Q[7] (1.00 g, 0.86 mmol) were dissolved in ethylene glycol under stirring. Subsequently, sodium acetate (CH₃COONa, 4.80 g, 58.4 mmol) and sodium citrate (0.60 g, 2.33 mmol) were added, and the resulting mixture was vigorously stirred for 30 min to obtain a homogeneous solution. The mixture was then transferred into a 100 mL Teflon-lined stainless-steel autoclave, sealed, and heated at 200 °C for 10 h. After naturally cooling to room temperature, the resulting black precipitate was collected and washed repeatedly with ethanol and deionized water. The obtained product was denoted as Q[7]MNPs and dried under vacuum at 60 °C for 24 h.

### Experimental methods

Solution ^1^H NMR spectra were recorded at 400 MHz on a JEOL JNM-ECZ400S spectrometer. Prior to NMR measurements, adsorbent samples were preheated at 25 °C for 15 min to remove surface-adsorbed vapors. FT-IR spectra were collected on a Nicolet iS50 FT-IR spectrometer (Thermo Fisher Scientific) using the KBr pellet method over the range of 4000–500 cm^−1^. Samples of Q[7] and Q[7]@MNPs were finely ground and thoroughly mixed with KBr before analysis. PXRD patterns were recorded on a Bruker D8 Advance diffractometer using Cu K*α* radiation (*λ* = 0.15406 nm). The operating voltage and current were set to 40 kV and 40 mA, respectively. Data were collected over a 2θ range of 10–80° with a scanning rate of 10° min^−1^ and a step size of 0.02°. TGA was carried out on a Hitachi 200 thermogravimetric analyzer under a nitrogen atmosphere (20 mL min^−1^). Samples of Q[7] and Q[7]@MNPs were heated from room temperature to 800 °C at a heating rate of 10 °C min^−1^. Magnetic properties of Q[7]@MNPs were measured using a vibrating sample magnetometer (VSM, Lake Shore 8604) at room temperature with a maximum applied magnetic field of 1 T, and the magnetic hysteresis loop was obtained. TEM images were acquired on a JEOL JEM-F200 electron microscope. Samples of Q[7] and Q[7]@MNPs (5–10 mg) were ultrasonically dispersed in anhydrous ethanol for 15 min, and a drop of the resulting suspension was deposited onto a carbon-coated copper grid. The samples were examined after solvent evaporation. Solid-state ¹³C CP-MAS NMR spectra were recorded on a Bruker AVANCE NEO 400 WB spectrometer at a ¹³C resonance frequency of 100.63 MHz using a 4 mm ZrO₂ MAS triple-resonance probe. SEM images were acquired on a Hitachi SU8600 field-emission scanning electron microscope, and EDS was performed on the same instrument for elemental mapping analysis. XPS measurements were carried out on a Thermo K-Alpha multifunctional imaging electron spectrometer (Thermo Fisher Scientific) using a monochromatized Al K*α* X-ray source (hν = 1486.68 eV) operated at 12 kV and 6 mA. The analysis chamber pressure was maintained at 5 × 10⁻⁹ mbar. Spectra were acquired with a pass energy of 50 eV and a step size of 0.1 eV. All binding energies were calibrated with reference to the C 1 s peak at 284.80 eV for charge correction. Raman spectra were collected on a WITec alpha 300 R confocal Raman microspectrometer using a 633 nm visible laser as the excitation source with a laser power of 10 mW, a grating of 1200 g/mm, and a spectral scanning range of 200–3500 cm⁻¹ with a spectral resolution of ≤1.5 cm⁻¹. The integration time was 10 s per scan, and spectra were accumulated over 3 scans to eliminate baseline noise. Baseline correction was performed using adaptive polynomial fitting, and peak fitting was carried out using a Gaussian–Lorentzian composite function to precisely extract peak intensities and peak positions. All raw spectral data points were exported from the instrument as continuous wavenumber–intensity datasets in Excel format. Nitrogen adsorption–desorption isotherms were measured at 77 K using a Micromeritics ASAP 2460 surface area and porosity analyzer. The BET specific surface area was calculated from the adsorption branch in the relative pressure range of 0.05–0.30, and the pore size distribution was derived from the Barrett–Joyner–Halenda (BJH) method.

## Supplementary information

Supplementary Information

Description of Additional Supplementary Files

Supplementary Data 1

Supplementary Video 1

Transparent Peer Review file

## Data Availability

The data that support the findings of this study are available within the article and its Supplementary Information. Source data are present. Atomic coordinates for DFT experiments are provided as Supplementary Data 1.
