# Supplementary material for: Cooperative outer-surface recognition extends host–guest chemistry for high-capacity molecular separation beyond cavity inclusion
Source: Nat Commun. 2026 Oct 8;17:11465. doi: 10.1038/s41467-026-78531-8 (PMC13740299; doi:10.1038/s41467-026-78531-8)
Supplement: Supplementary file 1 [file 41467_2026_78531_MOESM2_ESM.pdf]

File name: Supplementary Data 1.xlsx

Title: Cartesian coordinates of the DFT-optimized molecular structures.

Description:

This Excel file contains one worksheet, entitled “Atomic coordinates”, providing the Cartesian coordinates (in Å) of all DFT-optimized molecular structures examined in this study. The coordinate sets are arranged in separate blocks divided by blank rows. Each block lists the atom number, element symbol, and corresponding *x*, *y*, and *z* coordinates. The structures include pristine Q[7], isolated benzene (Ben), isolated *n*-heptane (*n*-Hep), and the Q[7]–Ben and Q[7]–*n*-Hep complexes representing the cavity-inclusion and outer-surface-binding configurations discussed in the main text and Fig. 6.

File name: Supplementary Video 1.avi

Title: Magnetic response and separation of Q[7]@MNPs under an external magnetic field.

Description:

This video shows the rapid response of dispersed Q[7]@MNPs to an external magnetic field. Upon placement of an external magnet near the dispersion, the nanoparticles migrate toward the magnet and form macroscopic chain-like aggregates, enabling their rapid magnetic separation from the liquid phase. This observation complements the magnetic characterization and separation results presented in Fig. 2i and Fig. S2.
