# Supplementary material for: Cooperative outer-surface recognition extends host–guest chemistry for high-capacity molecular separation beyond cavity inclusion
Source: Nat Commun. 2026 Oct 8;17:11465. doi: 10.1038/s41467-026-78531-8 (PMC13740299; doi:10.1038/s41467-026-78531-8)
Supplement: Supplementary file 2 [file 41467_2026_78531_MOESM5_ESM.pdf]

nature portfolio

Peer Review File

Cooperative Outer-Surface Recognition Extends Host–Guest Chemistry for High-Capacity Molecular Separation beyond Cavity Inclusion

Corresponding Author: Professor Xi-Sheng Wang

This file contains all reviewer reports in order by version, followed by all author rebuttals in order by version.

Version 0:

Reviewer comments:

Reviewer #1

(Remarks to the Author)
In this manuscript, Wang and coworkers report the immobilization of cucurbit[7]uril (Q[7]) onto magnetic $Fe3O4$ nanoparticles for the separation of benzene (Ben) from n-heptane (n-Hep). The authors propose that in addition to classical cavity-based inclusion, the outer surface of Q[7] can engage aromatic guests through cooperative π···π and C-H···π interactions, leading to a “cascade adsorption” process. The Q[7]@MNPs material exhibits high selectivity, enhanced adsorption capacity, and good recyclability. The concept of outer-surface-mediated recognition is presented as a general extension of host-guest chemistry. While the manuscript addresses a reasonably well-characterized material, the conceptual advance and mechanistic insights fall short of the high-impact standards expected for Nature Communications. Therefore, this work should be published in a journal with less stringent requirements on novelty and significance.
1. The outer-surface adsorption mechanism is not directly evidenced. The authors claim that Q[7] outer walls engage benzene molecules through π···π and C–H···π interactions, and that adsorbed benzene further recruits additional benzene molecules via aromatic stacking. However, no direct spectroscopic or structural evidence is provided to support this claim.
2. The immobilization of cucurbiturils on magnetic $Fe3O4$ nanoparticles was well documented a decade ago. The authors should discuss any new chemistry introduced by this work, in particular the synthesis of the material.
3. The potential formation of Q[7] aggregates or supramolecular assemblies on the nanoparticle surface, which could introduce new adsorption sites, is not investigated.
4. The authors don’t provide BET surface area analysis or pore-size distribution data to characterize the adsorption environment.
5. The DFT calculations only compare the cavity binding energies of Ben@Q[7] and n-Hep@Q[7], which do not support the proposed outer-surface binding mechanism.

Reviewer #2

(Remarks to the Author)
The separation of azeotropic and structurally similar mixtures remains a fundamental challenge for conventional porous materials, which are often constrained by rigid pore architectures and limited molecular discrimination. Supramolecular host–guest recognition offers molecular–level selectivity but is typically restricted by low adsorption capacity arising from one-to-one or one-to-two cavity inclusion. In this nice manuscript, the authors introduce a conceptual extension of classical host–guest chemistry that moves beyond exclusive cavity-based recognition to exploit cooperative interactions at the outer surface of macrocyclic hosts. Using cucurbiturils as a model system, the authors demonstrate that the outer surface of macrocycles can serve as an amplifying recognition interface, where an initial binding event anchors guest molecules and subsequently recruits additional guests through cooperative intermolecular interactions, resulting in cascade adsorption. By immobilizing cucurbit[7]uril (Q[7]) onto magnetic Fe_3O_4 nanoparticles, aggregation is suppressed and both cavity- and outer-surface-mediated recognition are simultaneously activated. This strategy is validated through the separation of benzene (Ben) from n-heptane (n-Hep), a prototypical aromatic/aliphatic azeotropic system. The resulting Q[7]@MNPs material exhibits dramatically enhanced adsorption capacity, complete selectivity, and excellent recyclability. This work establishes outer-surface-mediated cooperative recognition as a general extension of host–guest chemistry and provides a supramolecular design principle for overcoming the selectivity–capacity trade-off in challenging molecular separations. The

results presented in this well-written manuscript has good novelty and scientific value. This manuscript can be published as it is.

Reviewer #3

(Remarks to the Author)
The manuscript describes the synthesis of superparamagnetic Fe3O4 nanoparticles functionalized with cucurbit[7]uril Q[7] and their remarkable ability to selectively capture benzene in azeotropic mixtures benzene/heptane. The surprisingly high saturation capacity of these nanoparticles is mainly attributed to the ability of benzene to form stacks between Q[7] units spread around the nanoparticle, rather than to the well-known formation of an inclusion complex within the cavity of Q[7]. This conclusion is well supported by the adsorption data. Overall, I found the concept introduced in this article very elegant, and the simplicity of this system makes it attractive for real-life applications. Therefore, I support publication of the manuscript in Nature Communications.

The weakest part of the manuscript relates the binding studies performed by NMR and supported by DFT calculations. The NMR titration is showed because it highlights that Q[7] preferentially binds benzene over heptane and the calculations confirm that benzene fits well the cavity of Q[7]. In my opinion, these experiments are not particularly relevant for several reasons.

First, the titrations are performed in D2O, where binding is primarily driven by the hydrophobic effect. Therefore, the titrations do accurately not reflect the binding behavior observed in azeotropic mixtures benzene/heptane.

Second, the ability of cucurbit[7]uril to accommodate aromatic units in its cavity is well-established. Therefore, neither the NMR titration nor the DFT models bring much novelty to the manuscript.

I would prefer to know (for example) whether benzene binds Q[7] dissolved in pure heptane. If no binding occurs, this experiment would indicate that benzene preferentially binds between the Q[7] units arranged on the surface of the nanoparticle. If binding occurs, this experiment would support the claim that benzene binds both in the cavity AND between the Q[7] units arranged on the surface of the nanoparticle.

Likewise, it would be interesting to use modelling to confirm that benzene favorably interacts with the outer surface of Q[7], rather than in its cavity.

That said, the study is of high quality and the manuscript is nicely written.

Version 1:

Reviewer comments:

Reviewer #2

(Remarks to the Author)
The review comments from both Reviewer 1 and Reviewer 3 were addressed appropriately and enoughly. Therefore, this revised manuscript can be published as it is now.

Reviewer #3

(Remarks to the Author)
The manuscript has been strengthened by the inclusion of the additional experiments.
The characterization of Ben@Q[7]@MNPs is now more convincing and the new data provide stronger evidence that benzene can interact through binding modes other than inclusion within the cucurbituril cavity. However, the current results do not unambiguously demonstrate the existence of cooperative interactions at the outer surface of the macrocyclic hosts, as proposed in Figure 1. I would recommend that the authors moderate their conclusions and present this mechanism as a plausible hypothesis that could account for the observed results, rather than as a definitive finding.
In addition, I suggest removing all experiments like the NMR titration experiments performed in water, which are not relevant to this study and do not provide meaningful support for the main conclusions of the manuscript.

Open Access This Peer Review File is licensed under a Creative Commons Attribution 4.0 International License, which permits use, sharing, adaptation, distribution and reproduction in any medium or format, as long as you give appropriate credit to the original author(s) and the source, provide a link to the Creative Commons license, and indicate if changes were made.
In cases where reviewers are anonymous, credit should be given to 'Anonymous Referee' and the source.
The images or other third party material in this Peer Review File are included in the article's Creative Commons license, unless indicated otherwise in a credit line to the material. If material is not included in the article's Creative Commons license and your intended use is not permitted by statutory regulation or exceeds the permitted use, you will need to obtain permission directly from the copyright holder.
To view a copy of this license, visit https://creativecommons.org/licenses/by/4.0/

Dear Reviewers,

We thank the reviewers for their thoughtful comments and constructive suggestions on our manuscript (NCOMMS-26-018422). We have carefully considered all the points raised and have performed additional experiments, analyses, and theoretical calculations to address them. Below we provide a point-by-point response. All changes in the revised manuscript have been highlighted in yellow.

Response to Reviewer #1

General comment:

"While the manuscript addresses a reasonably well-characterized material, the conceptual advance and mechanistic insights fall short of the high-impact standards expected for Nature Communications. Therefore, this work should be published in a journal with less stringent requirements on novelty and significance."

Our response:

We respectfully disagree with the reviewer's assessment of the novelty and significance. The concept of using the outer surface of cucurbit[*n*]urils (specifically Q[7]) for selective adsorption, rather than the well-explored cavity inclusion, represents a paradigm shift in host–guest chemistry applied to separation. Unlike previous reports that rely solely on cavity encapsulation, we demonstrate a "cascade adsorption" mechanism driven by outer-surface π···π and C–H···π interactions, leading to exceptionally high capacity and selectivity for benzene over heptane. This work not only provides a new design principle for functional materials but also challenges the conventional focus on supramolecular macrocycle cavities. To strengthen our claims, we have now added direct evidence for the outer-surface binding mechanism (see responses to specific points below). We believe that after these revisions, the manuscript meets the high-impact standards of *Nature Communications*.

1. "The outer-surface adsorption mechanism is not directly evidenced. The authors claim that Q[7] outer walls engage benzene molecules through π···π and C–H···π interactions, and that adsorbed benzene further recruits additional benzene molecules via aromatic stacking. However, no direct spectroscopic or structural evidence is provided to support this claim."

Our response:

We thank the reviewer for this important comment. We fully agree that the proposed outer-surface adsorption mechanism should be supported by direct spectroscopic or structural evidence. Accordingly, we have substantially strengthened the mechanistic characterization in the revised manuscript by adding **solid-state ^{13}C CP/MAS NMR, XPS, and Raman spectroscopy**, which together provide complementary evidence for outer-surface adsorption and the subsequent cooperative assembly of benzene molecules.

First, **solid-state ^{13}C CP/MAS NMR** directly distinguishes benzene molecules in different adsorption environments. Whereas pure benzene exhibits a single resonance at ***δ* = 128.54 ppm**, Ben@Q[7]@MNPs displays two distinct resonances at ***δ* = 127.30** and **128.34 ppm**. According to previous studies on cucurbituril host–guest systems (*CrystEngComm* **2014**, *16*, 3788; *J. Am. Chem. Soc.* **2017**, *139*, 8138; *Materials* **2012**, *6*, 47), these two resonances are assigned to cavity-included and exterior-surface-adsorbed benzene, respectively. These results indicate that benzene exists in

two distinct adsorption environments, providing direct structural evidence that cavity inclusion and outer-surface adsorption coexist in Q[7]@MNPs.

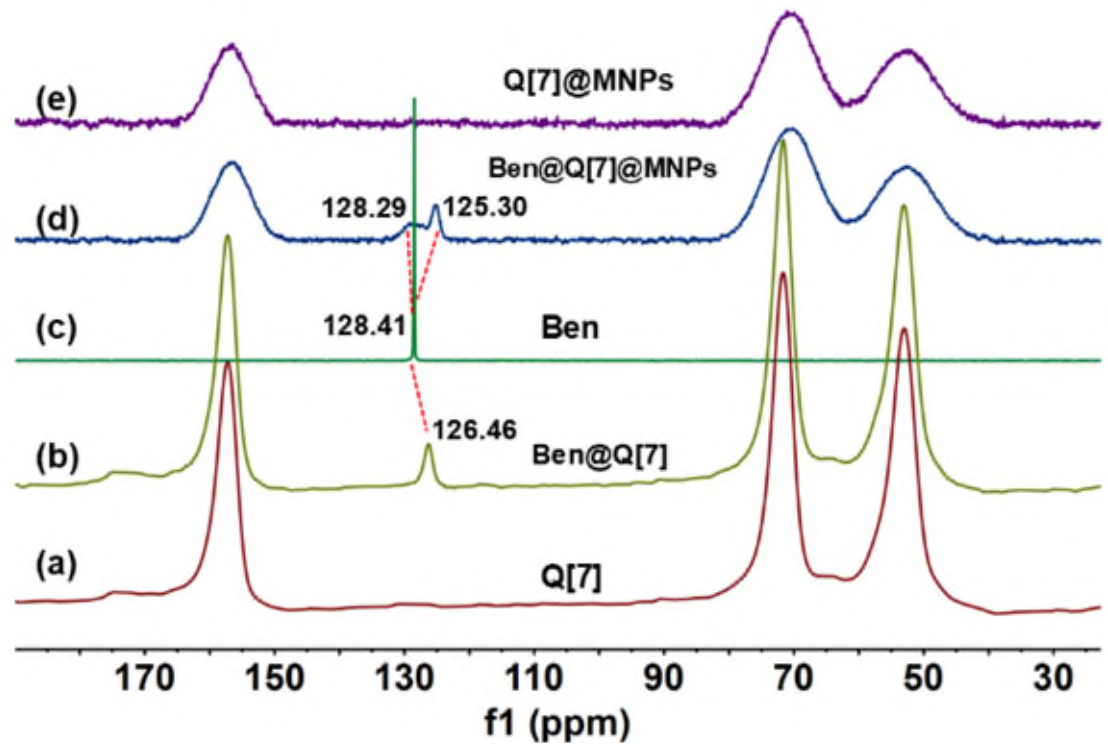

Solid-state ^{13}C CP/MAS NMR spectra of Q[7], Ben@Q[7], Ben, Ben@Q[7]@MNPs, and Q[7]@MNPs.

Second, **XPS** reveals the electronic interactions associated with exterior-surface adsorption. Compared with Q[7]@MNPs, Ben@Q[7]@MNPs exhibits a pronounced increase in the aromatic C=C component of the C 1s spectrum, whereas the Fe content and Fe 2p spectra remain essentially unchanged, indicating that the additional carbon originates from benzene adsorbed on Q[7] rather than on the Fe_3O_4 support. Moreover, both the N 1s and O 1s signals shift toward lower binding energies after benzene adsorption, consistent with electronic delocalization between the aromatic π system and the Q[7] framework (*Surf. Interfaces* **2025**, *66*, 106535; *Acc. Chem. Res.* **2014**, *47*, 1386). Because cavity inclusion is intrinsically limited to a 1:1 host–guest complex, whereas the observed XPS changes involve the overall surface chemical environment, these results are more consistent with extensive interactions occurring on the exposed outer surface of immobilized Q[7].

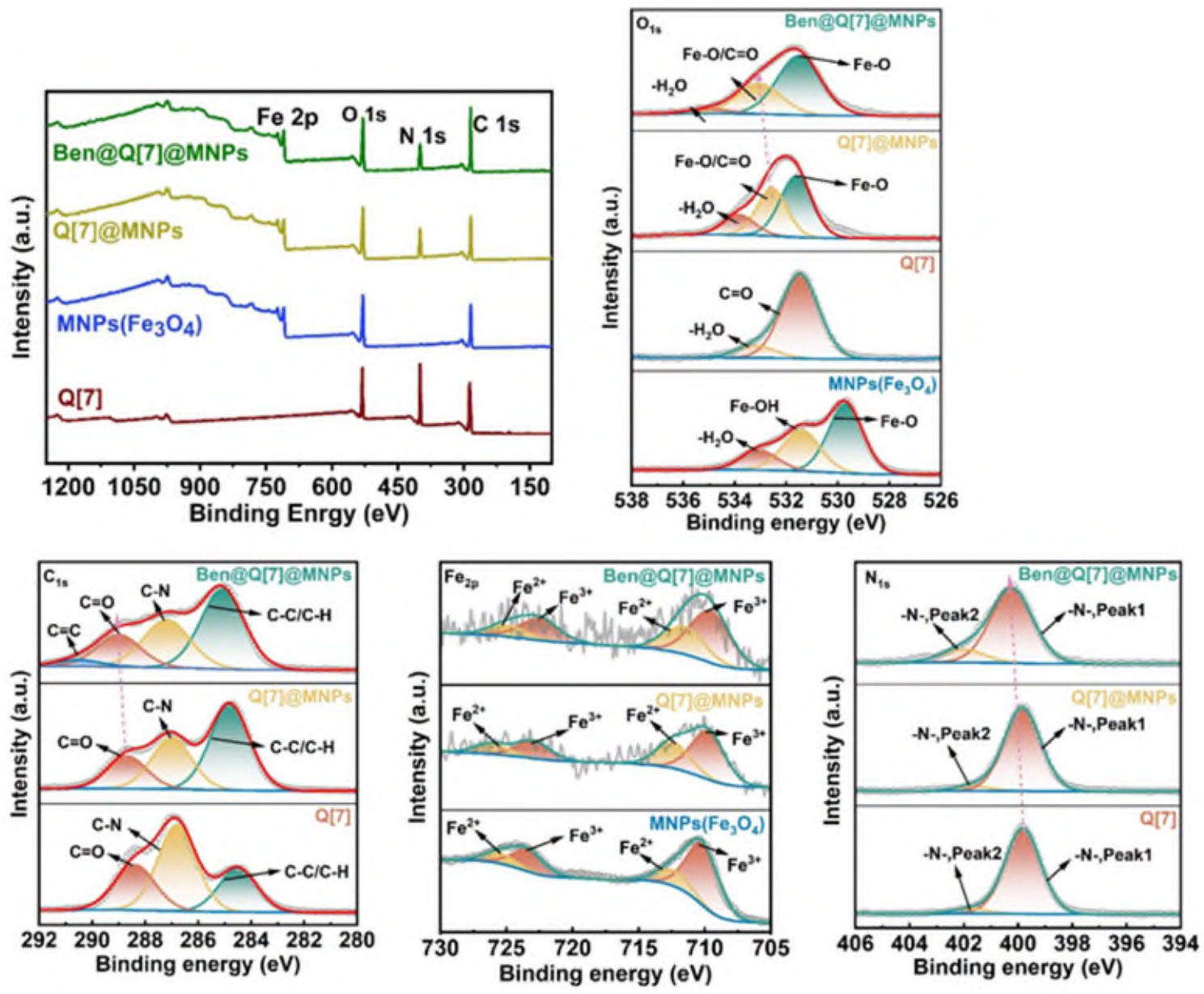

XPS survey spectra of Q[7], Fe_3O_4 MNPs, Q[7]@MNPs, and Ben@Q[7]@MNPs, and High-resolution XPS spectra of C 1s, Fe 2p, N 1s, and O 1s regions of the indicated samples.

Finally, **Raman spectroscopy** provides direct spectroscopic evidence for intermolecular aromatic stacking on the exterior surface (Figures 4h and S13, Tables S5 and S6). The characteristic breathing

and symmetric C–H stretching modes of benzene shift from **993 to 1001 cm^{-1}** and **3063 to 3069 cm^{-1}**, respectively, confirming cavity inclusion, in agreement with previous Raman studies of Q[7] host–guest complexes (*J. Phys. Chem. B* **2000**, *104*, 5084; *RSC Adv.* **2013**, *3*, 1354). More importantly, compared with Ben@Q[7], benzene adsorbed on Q[7]@MNPs exhibits substantially stronger Raman intensity, consistent with adsorption far exceeding the theoretical capacity permitted by cavity inclusion alone. Meanwhile, the Q[7] framework remains essentially unchanged, exhibiting only minor peak shifts and broadening (*Phys. Chem. Chem. Phys.* **2010**, *12*, 10429; *J. Phys. Chem. Lett.* **2016**, *7*, 704). Most importantly, the aromatic ν_8 C–C stretching mode and its associated Fermi resonance undergo pronounced red shifts (**1607 → 1596 cm^{-1}** and **1587 → 1570 cm^{-1}**, respectively), a well-established Raman signature of intermolecular π–π stacking (*Proteins: Struct. Funct. Bioinform.* **2013**, *81*, 690). These observations indicate that benzene molecules adsorbed on the outer surface further assemble through aromatic stacking, providing an additional driving force for cooperative adsorption.

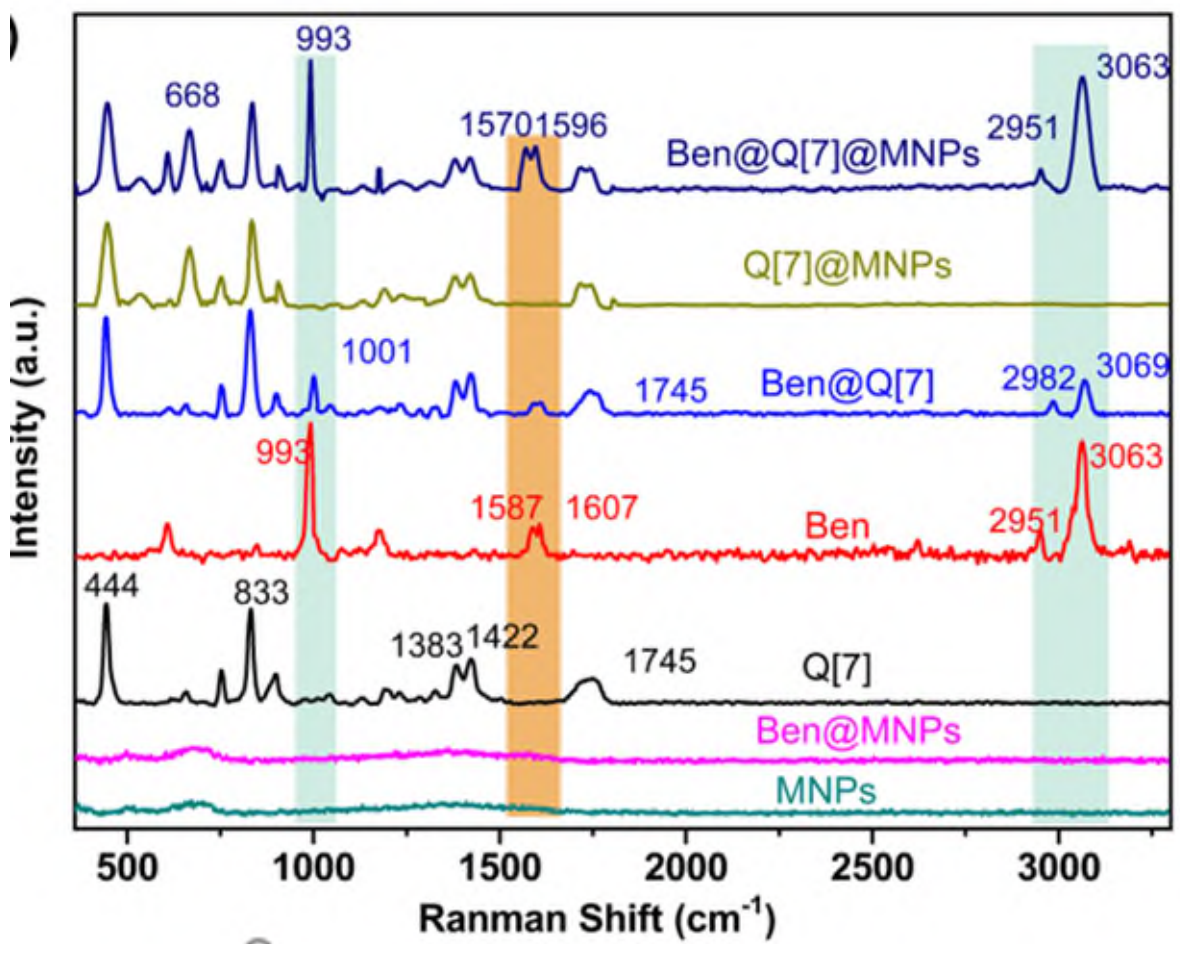

Raman spectra of Fe_3O_4 MNPs, Ben@MNPs, Q[7], Ben, Ben@Q[7], Q[7]@MNPs, and Ben@Q[7]@MNPs.

Collectively, the newly added **solid-state ^{13}C CP/MAS NMR, XPS, and Raman spectroscopy** establish a complementary mechanistic evidence chain: **solid-state NMR** distinguishes cavity inclusion from outer-surface adsorption, **XPS** identifies extensive electronic interactions between benzene and the exposed Q[7] framework while excluding significant adsorption on the Fe_3O_4 support, and **Raman spectroscopy** directly reveals intermolecular π–π stacking among exterior-surface-adsorbed benzene molecules. Together with the expanded DFT calculations (see our response to **Reviewer #2, Comment 5**), these results provide strong and mutually consistent support for the cooperative outer-surface adsorption mechanism proposed in the revised manuscript. The corresponding data and discussion have been incorporated into the revised manuscript and Supporting Information.

2. The immobilization of cucurbiturils on magnetic Fe_3O_4 nanoparticles was well documented a decade ago. The authors should discuss any new chemistry introduced by this work, in particular the synthesis of the material."

Our response:

We thank the reviewer for this insightful comment. In the revised manuscript, we have explicitly cited the previous report (*J. Mater. Chem. C* **2015**, *3*, 3517) on the immobilization of cucurbit[7]uril

(Q[7]) onto magnetic Fe_3O_4 nanoparticles in the section "Preparation of Q[7]@MNPs materials" (Q[7]@MNPs were synthesized according to a previously reported solvothermal procedure with minor modifications). We fully agree that the immobilization strategy itself is not unprecedented and do not claim synthetic novelty in simply anchoring Q[7] onto magnetic nanoparticles.

Instead, the key advance of the present work lies in the new supramolecular recognition behavior enabled by immobilization. Specifically, suppressing the aggregation of Q[7] through surface immobilization exposes not only the cavity binding sites but also the outer surface of the macrocycle, thereby enabling a cooperative recognition mechanism involving both cavity inclusion and outer-surface interactions. To the best of our knowledge, such synergistic adsorption behavior has neither been identified nor exploited in previous Q[7]-MNP systems.

Therefore, the novelty of this work does not reside in "how Q[7] is immobilized", but rather in "what new adsorption mechanism emerges after immobilization". The material platform allowed us to uncover an outer-surface-assisted cooperative recognition pathway that extends the conventional cavity-centered paradigm of cucurbituril-based separations.

3. The potential formation of Q[7] aggregates or supramolecular assemblies on the nanoparticle surface, which could introduce new adsorption sites, is not investigated."

Our response:

We thank the reviewer for raising this important point. To clarify whether Q[7] forms aggregates or supramolecular assemblies on the Fe_3O_4 surface, we carefully re-examined the structural characterization and further supplemented the manuscript with additional EDS analysis.

First, the TEM and HRTEM images provide direct structural evidence that Q[7] is uniformly dispersed rather than aggregated on the nanoparticle surface. As shown in the **Figure 1g,h**, Q[7]@MNPs exhibit a continuous patchy coating with an apparent thickness of approximately **1 nm**, which agrees well with the molecular height of a single Q[7] molecule (~0.91 nm). No multilayer coatings, large Q[7] aggregates, interparticle bridges, or interconnected supramolecular networks were observed throughout the sample. Instead, HRTEM reveals isolated Fe_3O_4 nanocrystallites surrounded by a thin amorphous interfacial layer, while the lattice spacing of **0.42 nm** remains clearly visible, indicating that the crystalline structure of Fe_3O_4 is preserved. These observations are therefore more consistent with a monolayer-like distribution of individually immobilized Q[7] molecules than with the formation of supramolecular assemblies. The observed morphology is also consistent with previous reports on cucurbituril-functionalized magnetic nanoparticles (*J. Mater. Chem. C* **2015**, *3*, 3517).

To further verify the spatial distribution of Q[7], we additionally performed EDS elemental mapping (Figure S1). The characteristic C and N signals originating from Q[7] are homogeneously distributed over the Fe_3O_4 nanoparticles without localized enrichment, providing further evidence against the presence of Q[7] aggregates.

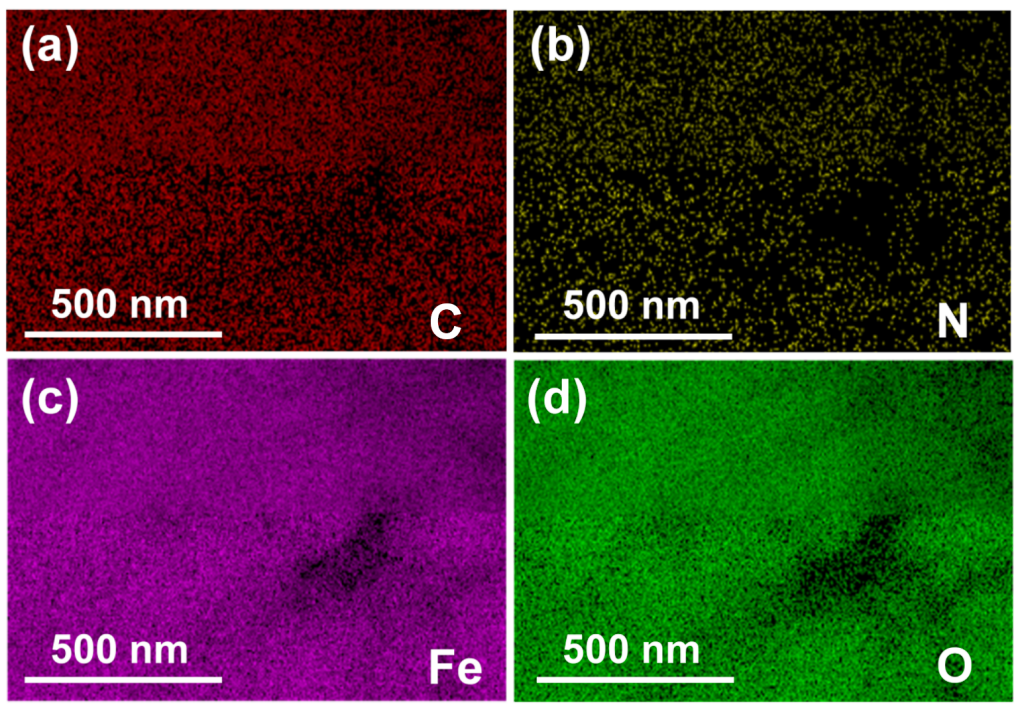

Figure S1. Elemental mapping images of Q[7]@MNPs.

The XRD (**Figure 1b**) and TGA (**Figure 1c**) results independently support this conclusion. No diffraction peaks attributable to crystalline Q[7] were detected in the XRD pattern of Q[7]@MNPs, indicating the absence of phase-separated or highly ordered Q[7] domains. Moreover, the relatively low Q[7] loading determined by TGA (8.70 wt%) is consistent with partial surface functionalization rather than multilayer accumulation.

Taken together, these complementary characterization results provide **no evidence** for the formation of Q[7] aggregates or supramolecular assemblies on the nanoparticle surface. Instead, they consistently support a model in which Q[7] molecules are individually immobilized as a dispersed interfacial layer. Consequently, the enhanced adsorption performance is attributed to the exposed cavities and outer surfaces of isolated Q[7] molecules, rather than to additional adsorption sites generated by interstitial voids within Q[7] aggregates or supramolecular assemblies.
Accordingly, the necessary relevant characterization data and discussion have been added to the revised **"Characterization of adsorbent"** section.

4. The authors don't provide BET surface area analysis or pore-size distribution data to characterize the adsorption environment."

Our response:

We thank the reviewer for this valuable suggestion. We agree that characterization of the surface area and pore structure is important for distinguishing conventional pore-controlled adsorption from supramolecular recognition-driven adsorption.

Following the reviewer's recommendation, we performed N_2 adsorption–desorption measurements on Q[7]@MNPs and have added the BET surface area and pore-size distribution data to the revised manuscript (Figure S3 and Table S2). The adsorption–desorption isotherm exhibits a typical type IV profile with an H3 hysteresis loop, indicating the presence of mesopores. Based on the adsorption isotherm collected at 77 K, the Brunauer–Emmett–Teller (BET) specific surface area was determined to be **16.44 m^2 g^{-1}**, with a narrow pore size distribution centered at approximately 3.6 nm.

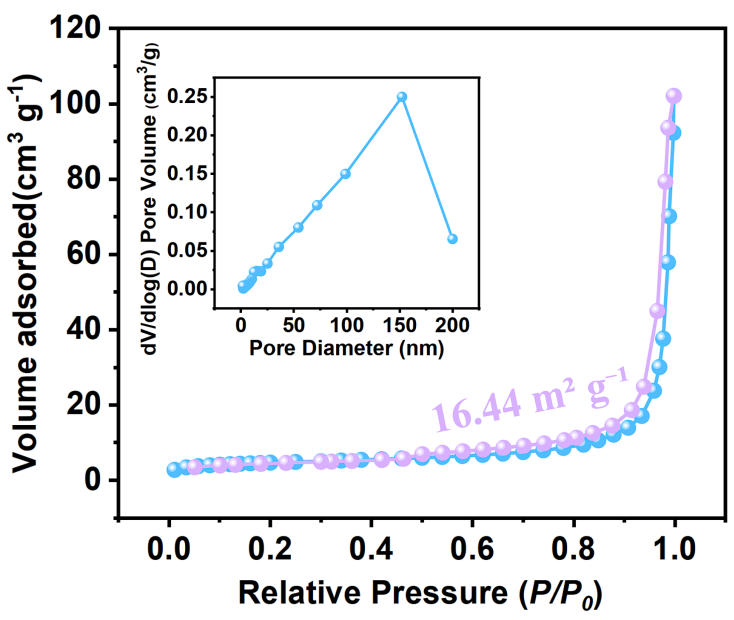

Figure S3. N_2 adsorption–desorption isotherm of Q[7]@MNPs at 77 K. Inset: BJH pore size distribution profile derived from adsorption branch data.

These results indicate that Q[7]@MNPs does not possess the high specific surface area or well-defined microporous architecture characteristic of conventional porous adsorbents. Therefore, the exceptional benzene uptake and selectivity are unlikely to arise primarily from surface-area or pore-confinement effects. Instead, the BET results support our proposed mechanism that adsorption is dominated by the molecular recognition properties of immobilized Q[7], which are further amplified through the cooperative cavity inclusion and outer-surface adsorption mechanism.

The corresponding BET isotherms, pore-size distribution, and the related discussion have been incorporated into the revised manuscript (Figure S1, Table S2, and the **"Characterization of adsorbent"** section).

5. The DFT calculations only compare the cavity binding energies of Ben@Q[7] and n-Hep@Q[7], which do not support the proposed outer-surface binding mechanism."

Our response:

We thank the reviewer for this insightful comment. We agree that the DFT calculations in the original manuscript, which only compared the cavity binding energies of Ben@Q[7] and *n*-Hep@Q[7], were insufficient to substantiate the proposed outer-surface adsorption mechanism. Accordingly, we have substantially expanded the computational study in the revised manuscript to explicitly investigate outer-surface adsorption and cooperative adsorption configurations.

First, the revised calculations further confirm the intrinsic preference of Q[7] for benzene within its cavity. The calculated binding energies of Ben@Q[7] and *n*-Hep@Q[7] are **−65.03** and **−53.08 kJ mol^{-1}** (Figure 5a), respectively, consistent with the competitive NMR experiments and the experimentally observed preferential adsorption of benzene.

More importantly, we now explicitly evaluated adsorption on the exterior surface of Q[7] (Figure 5b). A single benzene molecule adsorbed on the carbonyl-rich outer surface exhibits a favorable adsorption energy of **−34.85 kJ mol^{-1}**, whereas *n*-Hep interacts more weakly (**−31.87 kJ mol^{-1}**). These calculations demonstrate that the exposed outer surface of Q[7] is itself capable of preferentially recognizing aromatic guests, providing direct theoretical support for the proposed outer-surface adsorption pathway.

To examine whether cavity inclusion and outer-surface adsorption can occur simultaneously, we

further constructed a dual-site adsorption model containing one cavity-included benzene molecule together with a second benzene molecule adsorbed on the exterior surface (Figure 5c). The optimized structure remains thermodynamically stable (**E_{ads} = −27.89 kJ mol⁻¹**), indicating that the two adsorption modes can coexist.

Finally, to explore the origin of the exceptionally high adsorption capacity, we investigated cooperative adsorption models containing multiple benzene molecules on the exterior surface of Q[7] (Figure 5d). The calculated stabilization energies become progressively more favorable as the number of adsorbed benzene molecules increases (**−35.75**, **−37.22**, and **−44.75 kJ mol⁻¹** for two-, three-, and four-molecule assemblies, respectively), indicating that intermolecular aromatic stacking provides an additional thermodynamic driving force for cooperative adsorption.

Taken together, the revised DFT calculations extend well beyond the cavity-binding comparison presented in the original manuscript and directly address the reviewer's concern. The computational results support three key conclusions: **(i)** the exterior surface of Q[7] provides thermodynamically favorable adsorption sites for benzene; **(ii)** cavity inclusion and outer-surface adsorption can coexist; and **(iii)** cooperative adsorption mediated by intermolecular aromatic stacking further stabilizes benzene assemblies on the Q[7] surface. These new calculations have been incorporated into the revised manuscript (Figure 5) and provide theoretical support for the cooperative cavity inclusion/outer-surface adsorption mechanism proposed in this work.

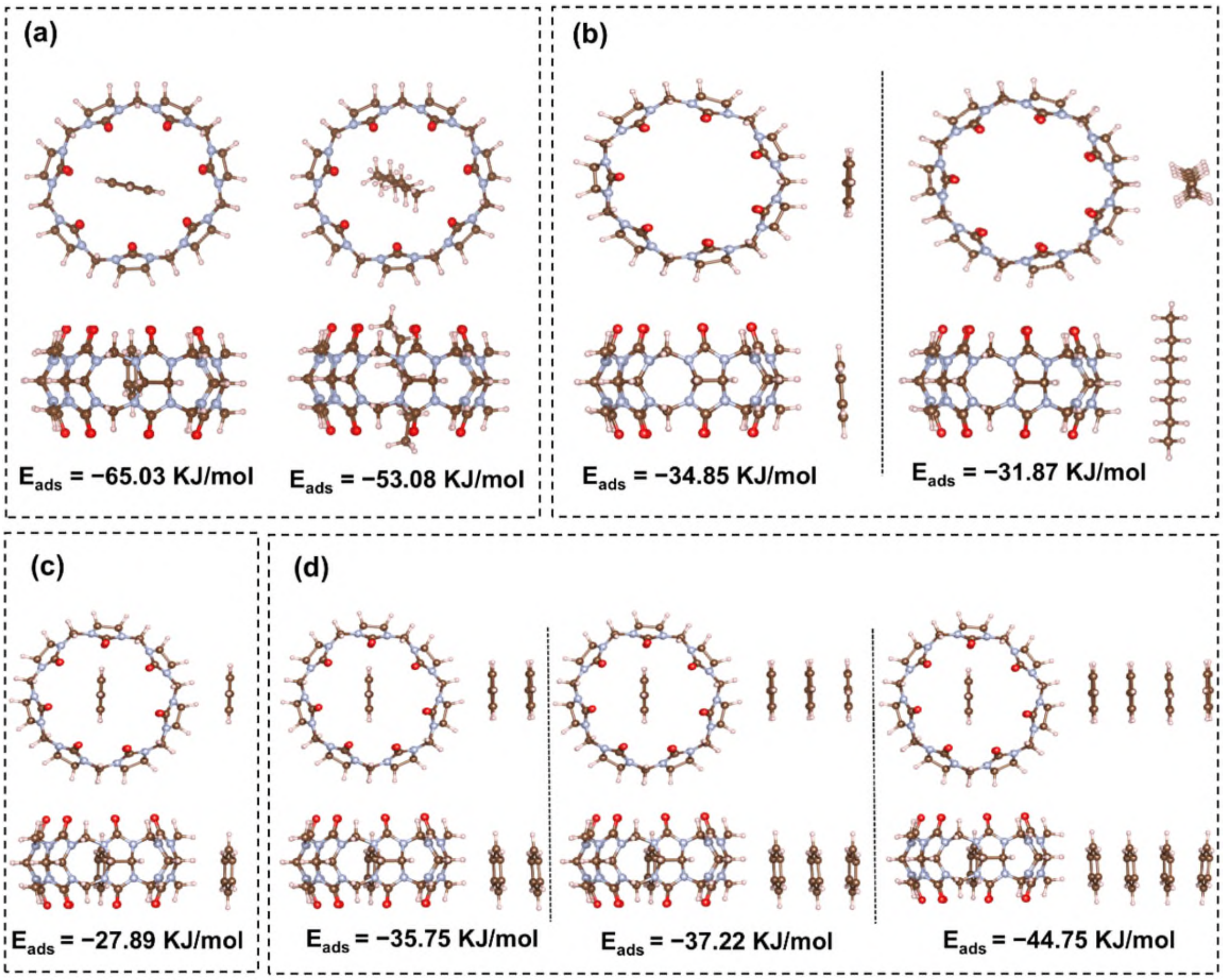

Figure 5. DFT calculations of adsorption mechanisms in the Q[7] system. (a) Cavity inclusion of Ben and *n*-Hep in Q[7]. (b) Outer-surface adsorption of Ben and *n*-Hep on Q[7]. (c) Dual-site adsorption involving simultaneous cavity

and surface binding of Ben. (d) Cooperative π–π stacking of multiple Ben molecules (n = 2–4) on the Q[7] surface, showing progressively enhanced stabilization.

Response to Reviewer #3

General comment:

"Overall, I found the concept introduced in this article very elegant, and the simplicity of this system makes it attractive for real-life applications. Therefore, I support publication of the manuscript in Nature Communications."

Our response:

We greatly appreciate the reviewer's positive assessment and strong support for publication. We have carefully addressed the specific concerns regarding the NMR and DFT studies, as detailed below.

1. The titrations are performed in D_2O, where binding is primarily driven by the hydrophobic effect. Therefore, the titrations do accurately not reflect the binding behavior observed in azeotropic mixtures benzene/heptane."

Response:

We thank the reviewer for this important comment. We fully agree that the binding behavior observed by solution-state NMR in D_2O is primarily driven by the hydrophobic effect and therefore cannot be quantitatively extrapolated to the Ben/*n*-Hep azeotropic system.

We would like to clarify, however, that this was not the purpose of including the solution-state NMR experiments in the original manuscript. Rather, these experiments were designed solely to establish the **intrinsic molecular recognition capability of the Q[7] cavity**, namely that Q[7] preferentially recognizes Ben over *n*-Hep when both guests compete for the same binding site. This represents the molecular basis for the subsequent investigation of the adsorption mechanism of immobilized Q[7]@MNPs, rather than an attempt to reproduce the thermodynamics of the nonpolar adsorption system.

Using an aqueous model system is both experimentally necessary and well established in cucurbituril chemistry. Because Q[7] is essentially insoluble in *n*-Hep and Ben/*n*-heptane mixtures, direct solution-state binding measurements under the adsorption conditions proposed by the reviewer are experimentally infeasible. Consequently, aqueous NMR and competitive binding experiments have long served as the standard approach for probing the intrinsic host–guest recognition properties of cucurbiturils toward aromatic and aliphatic hydrocarbons (*J. Am. Chem. Soc.* **2006**, *128*, 14871; *J. Am. Chem. Soc.* **2012**, *134*, 15318; *Angew. Chem. Int. Ed.* **2011**, *50*, 9338; *Chem* **2020**, *6*, 1082).

More importantly, **our mechanistic conclusions do not rely solely on the aqueous NMR experiments**. In the revised manuscript, we have substantially strengthened the evidence obtained directly under, or closely related to, the adsorption conditions. Specifically, (i) static adsorption experiments using solid Q[7] exposed to Ben/*n*-heptane vapor demonstrate a Ben selectivity of 98.83%, directly confirming preferential Ben adsorption in a nonaqueous environment; (ii) cavity-blocking experiments using a high-affinity competitive guest show that Ben adsorption is almost

completely suppressed when the Q[7] cavity is occupied, confirming that cavity inclusion is the primary recognition pathway; and (iii) solid-state ^{13}C CP/MAS NMR, XPS, Raman spectroscopy, and expanded DFT calculations further demonstrate that, in immobilized Q[7]@MNPs, cavity inclusion is complemented by cooperative outer-surface adsorption, accounting for the experimentally observed adsorption capacity far beyond the theoretical 1:1 cavity limit.

To avoid potential misunderstanding, we have revised the corresponding discussion in the manuscript to explicitly state that the aqueous NMR experiments are intended only to probe the intrinsic host–guest recognition properties of Q[7], rather than to quantitatively mimic the adsorption thermodynamics in the Ben/*n*-heptane system. The adsorption mechanism proposed in this work is instead established from the combined evidence of nonaqueous adsorption experiments, solid-state spectroscopic characterization, and DFT calculations.

We thank the reviewer for this valuable comment, which has helped us clarify both the scope and the limitations of the solution-state NMR experiments in the revised manuscript.

2. The ability of cucurbit[7]uril to accommodate aromatic units in its cavity is well-established. Therefore, neither the NMR titration nor the DFT models bring much novelty to the manuscript.”

Our response:

We sincerely thank the reviewer for this insightful comment. We fully agree that the ability of cucurbit[7]uril (Q[7]) to encapsulate aromatic guests within its cavity has been well established. Accordingly, the solution-state NMR titration and the original cavity-binding DFT calculations were included as mechanistic reference experiments rather than as claims of conceptual novelty.

Establishing the intrinsic preference of the Q[7] cavity for benzene over *n*-heptane is nevertheless essential, as it provides the mechanistic baseline against which the additional adsorption pathway introduced by immobilization can be distinguished. Without this reference, it would not be possible to separate the contribution of classical cavity inclusion from the enhanced adsorption arising from immobilized Q[7]@MNPs.

More importantly, the revised manuscript no longer relies on the well-established cavity inclusion chemistry as the principal mechanistic evidence. Instead, we have substantially expanded the mechanistic investigation to directly probe the newly proposed outer-surface adsorption pathway by combining solid-state **^{13}C CP/MAS NMR**, XPS, Raman spectroscopy, and expanded DFT calculations (see also our response to Reviewer #1, Comment 5). The new DFT calculations demonstrate that, although cavity inclusion remains thermodynamically preferred (−65.03 kJ mol^{-1}), benzene also binds favorably to the carbonyl-rich outer surface of Q[7] (−34.85 kJ mol^{-1}). Furthermore, dual-site and multi-benzene adsorption models reveal that cavity inclusion, outer-surface adsorption, and intermolecular aromatic stacking can coexist and cooperatively stabilize the adsorbed state. These theoretical results are fully consistent with the newly added spectroscopic evidence and directly support the cooperative adsorption mechanism proposed in this work.

We therefore agree that cavity inclusion by Q[7] is established chemistry and is not the conceptual advance of this study. Rather, the conceptual advance lies in demonstrating that immobilization

transforms Q[7] from a conventional single-site host into a dual-site recognition platform, in which classical cavity inclusion operates cooperatively with a newly accessible outer-surface adsorption pathway to produce adsorption capacities far beyond the intrinsic 1:1 host–guest limit while maintaining exceptional benzene selectivity. We have revised the manuscript throughout to make this distinction explicit.

3. "I would prefer to know (for example) whether benzene binds Q[7] dissolved in pure heptane. If no binding occurs, this experiment would indicate that benzene preferentially binds between the Q[7] units arranged on the surface of the nanoparticle. If binding occurs, this experiment would support the claim that benzene binds both in the cavity AND between the Q[7] units arranged on the surface of the nanoparticle. "

Our response:

We thank the reviewer for this thoughtful suggestion. We agree that directly probing the interaction between benzene and molecularly dissolved Q[7] in pure *n*-heptane would, in principle, provide valuable information for distinguishing cavity inclusion from adsorption associated with immobilized Q[7] on the nanoparticle surface.

Unfortunately, this experiment is not experimentally feasible because Q[7] is essentially insoluble in *n*-heptane and other nonpolar hydrocarbon solvents. Consequently, the proposed solution-state binding experiment cannot be performed under the suggested conditions.

Although this direct experiment is not accessible, several independent experimental and computational results collectively address the same mechanistic question. First, solution-state NMR, competitive binding experiments, and cavity-blocking experiments consistently demonstrate that benzene is preferentially encapsulated within the Q[7] cavity. In particular, preoccupation of the cavity by a strong competitive guest almost completely suppresses benzene adsorption by free Q[7], confirming that cavity inclusion is an intrinsic recognition pathway.

However, cavity inclusion alone cannot account for the adsorption behavior of Q[7]@MNPs. Based on the Q[7] loading, the theoretical adsorption capacity arising solely from 1:1 cavity inclusion is only **5.84 mg g^{-1}**, whereas the experimentally observed capacity reaches **136.17 mg g^{-1}**, indicating that additional adsorption sites must contribute to benzene uptake.

To directly investigate these additional adsorption pathways, we have substantially strengthened the mechanistic characterization in the revised manuscript. Solid-state **^{13}C CP/MAS NMR** reveals two distinct adsorption environments for benzene, consistent with cavity-included and exterior-surface-adsorbed species. **XPS** further indicates extensive interactions between benzene and the exposed Q[7] framework, while **Raman spectroscopy** provides spectroscopic evidence for intermolecular π–π stacking between benzene molecules adsorbed on the exterior surface. In parallel, the expanded **DFT calculations** demonstrate that adsorption on the outer surface of Q[7] is thermodynamically favorable, that cavity inclusion and outer-surface adsorption can coexist, and that cooperative adsorption through aromatic stacking is energetically feasible.

Collectively, although the experiment suggested by the reviewer cannot be carried out because of

the intrinsic insolubility of Q[7] in *n*-heptane, the complementary evidence obtained from solution-state NMR, solid-state NMR, XPS, Raman spectroscopy, adsorption measurements, and DFT calculations consistently supports a dual-pathway adsorption mechanism. Specifically, benzene is initially recognized through cavity inclusion and is further enriched through cooperative adsorption on the exposed outer surface of immobilized Q[7]. We have incorporated these additional experimental data, computational results, and mechanistic discussion into the revised manuscript.

4. "It would be interesting to use modelling to confirm that benzene favorably interacts with the outer surface of Q[7], rather than in its cavity."

Our response:

We thank the reviewer for this valuable suggestion. In response, we have substantially expanded the DFT calculations to explicitly investigate benzene adsorption on the exterior surface of Q[7], in addition to the conventional cavity inclusion model (see our response to **Reviewer #1, Point 5** for details).

Specifically, the revised calculations demonstrate that adsorption of benzene on the outer surface of Q[7] is thermodynamically favorable, that cavity inclusion and outer-surface adsorption can coexist within the same system, and that cooperative adsorption involving multiple benzene molecules is also energetically feasible. These calculations provide direct theoretical support for the proposed outer-surface adsorption mechanism and complement the newly added solid-state NMR, XPS, and Raman spectroscopic evidence.

The corresponding computational results and discussion have been incorporated into the revised manuscript (Figure 5).

We believe that the revised manuscript has been substantially strengthened through these revisions and now provides a comprehensive and well-supported mechanistic understanding of the cooperative adsorption mechanism. We sincerely thank the reviewers for their thoughtful and constructive comments, which have significantly improved the quality and clarity of this manuscript.

Sincerely,

Qing Li and Xi-Sheng Wang

Manuscript ID: NCOMMS-26-018422A

Title: Cooperative Outer-Surface Recognition Extends Host–Guest Chemistry for High-Capacity Molecular Separation beyond Cavity Inclusion

Dear Reviewers,

We sincerely thank the Reviewers for their careful evaluation of our manuscript and for the constructive suggestions provided during the review process. We greatly appreciate the insightful comments, which have helped us improve the clarity, rigor, and overall quality of our manuscript.

In this second-round revision, we have carefully considered all remaining concerns raised by the reviewers and have revised the manuscript accordingly. In particular, following the suggestions from Reviewer #3, we have moderated the mechanistic interpretation of outer-surface-mediated recognition and revised the corresponding descriptions throughout the manuscript to avoid overstating the conclusions. Furthermore, we have removed the solution-state NMR titration experiments performed in water, as suggested by the reviewer, and streamlined the mechanistic discussion to better focus on the evidence directly relevant to adsorption beyond cavity inclusion. All revisions are highlighted in the revised manuscript, and detailed responses to each comment are provided below.

We sincerely appreciate the reviewers' valuable suggestions, which have significantly improved the scientific rigor and presentation of our work.

Response to Reviewer #3

Comment 1: "The manuscript has been strengthened by the inclusion of the additional experiments. The characterization of Ben@Q[7]@MNPs is now more convincing and the new data provide stronger evidence that benzene can interact through binding modes other than inclusion within the cucurbituril cavity. However, the current results do not unambiguously demonstrate the existence of cooperative interactions at the outer surface of the macrocyclic hosts, as proposed in Figure 1. I would recommend that the authors moderate their conclusions and present this mechanism as a plausible hypothesis that could account for the observed results, rather than as a definitive finding."

Our response:

We sincerely thank the reviewer for this valuable comment and appreciate the recognition that the additional experiments have strengthened the characterization of Ben@Q[7]@MNPs.

We fully agree with the reviewer that the mechanistic interpretation should be presented with appropriate caution. In the revised manuscript, we have therefore moderated our conclusions and no longer describe the outer-surface-mediated cooperative recognition process as an unequivocally established mechanism. Instead, we now present it as a **plausible mechanistic model supported by multiple experimental observations and theoretical calculations**.

Specifically, we have carefully revised the relevant descriptions throughout the manuscript. Statements implying definitive proof of outer-surface cooperative recognition have been replaced

with more appropriate expressions, including “suggest,” “support,” and “are consistent with.” The proposed mechanism is now described as a hierarchical adsorption model, in which classical cavity inclusion and additional exterior-associated interactions may collectively contribute to the enhanced adsorption performance.

Furthermore, the corresponding discussion has been revised to clarify that the experimental results provide evidence for adsorption pathways beyond cavity inclusion, while the detailed nature of the proposed cooperative interactions remains a mechanistic interpretation supported by converging evidence.

We appreciate the reviewer’s suggestion, which has helped us achieve a more balanced and scientifically rigorous presentation of the proposed mechanism.

Comment 2: “In addition, I suggest removing all experiments like the NMR titration experiments performed in water, which are not relevant to this study and do not provide meaningful support for the main conclusions of the manuscript.”

Our response:

We sincerely thank the reviewer for this suggestion. We agree that the solution-state NMR titration experiments mainly describe the well-established cavity inclusion behavior of Q[7] and do not directly provide evidence for the proposed outer-surface interactions. Therefore, following the reviewer’s recommendation, we have removed these experiments and the corresponding discussion from the revised manuscript. The revised manuscript now focuses on the experimental and theoretical evidence directly related to adsorption beyond classical cavity inclusion, including adsorption capacity analysis, solid-state characterization, surface chemical analysis, Raman spectroscopy, and density functional theory calculations. By removing the solution-state NMR discussion, the revised manuscript provides a more focused presentation of the central concept and avoids overemphasizing the conventional host–guest inclusion process.

We sincerely appreciate the reviewer’s constructive suggestion, which has improved the focus and clarity of the revised manuscript.

Sincerely,

Qing Li and Xi-Sheng Wang
