# Supplementary material for: Cooperative outer-surface recognition extends host–guest chemistry for high-capacity molecular separation beyond cavity inclusion
Source: Nat Commun. 2026 Oct 8;17:11465. doi: 10.1038/s41467-026-78531-8 (PMC13740299; doi:10.1038/s41467-026-78531-8)
Supplement: Supplementary file 3 [file 41467_2026_78531_MOESM1_ESM.docx]

**Supplementary Information**

**Cooperative Outer-Surface Recognition Extends Host–Guest Chemistry for High-Capacity Molecular Separation beyond Cavity Inclusion**

Yafang Zhang,^1^ Tao Hou,^1^ Lixia Chen,^1^ Zhu Tao,^1^ Xi-Sheng Wang,^1,2^* and Qing Li^1^*

1 School of Chemistry and Chemical Engineering, State Key Laboratory of Green Pesticide, Guizhou University, Guiyang 550025, China.

2 Hefei National Research Center for Physical Sciences at the Microscale and Department of Chemistry, University of Science and Technology of China, Hefei 230026, China.

*Corresponding Authors. Q. Li: liq@gzu.edu.cn; X.-S. Wang: xswang77@ustc.edu.cn

**Table of Contents**

1. Methods………………………………………………………………….…...S3

2. Characterization of Q[7]@MNPs……………………………….…….….......S5

3. Uptake from Ben/*n*-Hep mixtures by bare Fe₃O₄, Q[7], and Q[7]@MNPs......S8

4. Adsorption kinetic and isotherm studies in adsorption of Ben……..……..…S11

5. Raman spectroscopic analysis……….…………………………………….…S12

6. Cycling performance………………….....…………………………………...S14

7. Purification of *n*-Hep……………………………………………………...….S16

8. References………………………………………………………………..…...S17

**1.** **Methods**

**1.1 Gas chromatography**

Gas chromatography (GC) measurements were carried out on a SHIMADZU GC-2014C system equipped with a flame ionization detector (FID) and an SE-30 capillary column (60 m × 0.32 mm i.d., 1.0 μm film thickness). Samples were analyzed by headspace injection. Briefly, sealed vials were heated at 70 °C for 15 min to establish vapor–liquid equilibrium, and 1.0 mL of the headspace gas was subsequently injected. The oven temperature was programmed from 50 °C to 120 °C at a heating rate of 10 °C min^−1^ and held at 120 °C for 5 min, giving a total run time of 15 min. The injector and detector temperatures were set at 250 °C and 280 °C, respectively. Nitrogen was used as the carrier gas at a flow rate of 1 mL min^−1^, while the FID was supplied with hydrogen (40 mL min^−1^) and air (300 mL min^−1^). A split ratio of 50:1 was applied during injection.

**1.2 Adsorption experiments**

In order to explore the adsorption performance of Q[7]@MNPs for benzene, at 25 ℃, the volume is 15 mL, the initial benzene concentration (C_0_) is prepared, the dosage of Q[7]@MNPs is 100 mg, at 400 rpm, and the equilibrium adsorption experiment is carried out at room temperature for 120min. Finally, the adsorbent is separated from the solution with a permanent magnet, and the equilibrium concentration of benzene is determined by a gas chromatograph. The formula for calculating the adsorption amount (1) is as follows:

$q_{t}=\frac{\left( C_{0}-C_{e} \right)V}{m}$	(1)

*q_t_* (mg/g) is the unit mass adsorption, *C*_0_ and *C_e_* (mg/L) are the initial concentration and equilibrium concentration of benzene, m (g) is the mass of the adsorbent, and V is the volume of the adsorbent solution (L). Pseudo-first-order kinetic model (PFO) (formula 2) and Pseudo-second-order kinetic model (PSO) (formula 3) Fit the data of the adsorption equilibrium curve and calculate the relevant data.

$\mathrm{In}\left( q_{e}-q_{t} \right)=\mathrm{In}q_{e}-k_{1}t$	(2)

$\frac{t}{q_{t}}=\frac{t}{q_{e}}+\frac{1}{k_{2}q_{e}^{2}}$	(3)

Among them, *q_e_* (mg/g) is the adsorption amount (mg/g) at adsorption equilibrium, qt is the adsorption amount (mg/g) at time t, and k_1_ (min^-1^) and k_2_ (g/mg/min) are the rate constants of PFO and PSO models respectively. Introduce two commonly used isothermal adsorption models, Langmuir (formula 4) and Freundlich (formula 5), to fit and analyze the data and calculate the relevant parameters. The formula is as follows:

$\frac{C_{e}}{q_{e}}=\frac{1}{q_{m}K_{L}}+\frac{C_{e}}{q_{m}}$	(4)

$\mathrm{In}q_{e}=\mathrm{In}K_{F}+\frac{1}{n}\mathrm{In}C_{e}$	(5)

Among them, *C_e_* (mg/L) is the content of benzene at equilibrium, *q*_m_ (mg/g) is the theoretical maximum adsorption capacity, *K*_L_ (L/mg) and *K*_F_ (L/mg) are the adsorption equilibrium constants of the Langmuir isotherm model and the Freundlich isotherm model respectively, and n is the empirical constant of the Freundlich isotherm model.

**1.3 Density functional theory calculations**

All the DFT calculations were conducted based on the Vienna Ab initio Simulation Package (VASP).^[1,2]^ The exchange-correlation potential was described by the Perdew−Burke−Ernzerhof (PBE) generalized gradient approach (GGA).^[3]^ The electron-ion interactions were accounted by the projector augmented wave (PAW).^[4]^ All DFT calculations were performed with a cut-off energy of 400 eV, and the Brillouin zone was sampled using a 3 × 3 × 1 k-point grid. The energy and force convergence criteria of the self-consistent iteration were set to 10^−4^ eV and 0.05 eV Å^−1^, respectively. DFT-D3 method was used to describe van der Waals (vdW) interactions.^[5]^ VESTA software was used to visualize the results. The adsorption energy (*E*_ads_) is defined as:

$$E_{ads}=E_{complex}-E_{substrate}-E_{adsorbate}$$

where substrate and adsorbate refer to the substrate surface and adsorbate molecule, respectively.

**2. Characterization of Q[7]@MNPs**

**2.1 Elemental analysis**

**Table S1** The elemental analysis of Q[7]@MNPs

| Name | C | N | H |
| --- | --- | --- | --- |
| Mass percent (wt.%) | 6.058 | 2.933 | 1.572 |

**2.2 Elemental mapping**

**Fig. S1** Elemental mapping images of Q[7]@MNPs.

**2.3 Magnetic responsive performance**

As shown in Supplementary Video 1, a typical macroscopic chain-like structure characteristic of the superparamagnetic behavior of magnetic nanoparticles is formed upon adjusting the position of the external magnet.

**Fig.** **S2** Magnetic attraction behavior of Q[7]@MNPs in the presence of an external magnet.

**2.4 N₂ physisorption characterization**

**Fig. S3** The N_2_ adsorption − desorption isotherms (Inset is pore size distribution profiles) of Q[7]@MNPs.

**Table S2** Summary of surface area and pore volume parameters of Q[7]@MNPs derived from N₂ physisorption at 77 K.

| Parameter | Method | Q[7]@MNPs |
| --- | --- | --- |
| BET Surface Area (m²/g) | BET | 16.44 |
| Single Point Surface Area at p/p° ≈ 0.25 (m²/g) | Single Point | 16.17 |
| Langmuir Surface Area (m²/g) | Langmuir | 30.69 |
| t-Plot Micropore Area (m²/g) | t-Plot | 4.42 |
| t-Plot External Surface Area (m²/g) | t-Plot | 12.02 |
| BJH Adsorption Cumulative Pore Area (m²/g) | BJH (Ads) | 11.8 |
| BJH Desorption Cumulative Pore Area (m²/g) | BJH (Des) | 18.46 |
| Single Point Adsorption Total Pore Volume (cm³/g)ᵃ | p/p° = 0.995 | 0.123 |
| Single Point Desorption Total Pore Volume (cm³/g)ᵃ | p/p° = 0.995 | 0.155 |
| t-Plot Micropore Volume (cm³/g) | t-Plot | 0.0021 |
| BJH Adsorption Cumulative Pore Volume (cm³/g)ᵇ | BJH (Ads) | 0.14 |
| BJH Desorption Cumulative Pore Volume (cm³/g)ᵇ | BJH (Des) | 0.158 |

ᵃ Total pore volume obtained by single-point nitrogen adsorption at P/P₀ = 0.995.

ᵇ Cumulative mesopore volume (2–200 nm) calculated via the BJH model from the adsorption branch of the isotherm.

3. **Uptake from Ben/*n*-Hep mixtures by bare Fe₃O₄, Q[7], and Q[7]@MNPs**

**Fig. S4** Time-dependent solid–vapor adsorption plots of Fe_3_O_4_ for a mixed vapor of Ben and *n*-Hep (v/v = 1:1).

**Fig. S5** Gas chromatography analysis of the retention time of Ben.

**Fig. S6** Gas chromatography analysis of the retention time of *n*-Hep.

**Fig. S7** Relative uptake of the Ben/*n*-Hep mixture (v/v = 1:1) adsorbed by Q[7] in the solid–vapor phase using gas chromatography

**Fig. S8** Time-dependent solid–vapor adsorption of Ben on Q[7].

**Fig. S9** Relative uptake of the Ben/*n*-Hep mixture (v/v = 1:1) adsorbed by Q[7]@MNPs in the solid–vapor phase using gas chromatography.

**4. Adsorption kinetic and isotherm studies in adsorption of Ben**

**Table S3** Kinetic parameters for the adsorption of Ben on Q[7]@MNPs.

|  | Pseudo-First-order |  | Pseudo-second-order |
| --- | --- | --- | --- |
| *q*_e_,_exp_ (mg/g) | 164.57 | *q*_e_,_exp_ | 157.48 |
| R^2^ | 0.97 | R^2^ | 0.99 |
| K_1_ | 0.5434 | K_2_ | 0.0572 |
| *q*_e,cal_ (mg/g) |  | 136.17 |  |

**Table S4** Langmuir and Freundlich parameters for the adsorption of Ben on Q[7]@MNPs.

|  | Langmuir |  |  | Freundlich |  |
| --- | --- | --- | --- | --- | --- |
| *q*_m_ (mg/g) | *K*_L_ | R^2^ | n | *K*_F_ | R^2^ |
| 149.03 | 0.05497 | 0.99 | 3.9758 | 31.59 | 0.72 |

**Fig. S10** Adsorption kinetic curve of Q[7]@MNPs for Ben solution.

**5. Raman spectroscopic analysis**

**Fig. S11** Enlarged view of the stacked Raman spectra.

**Table S5** Raman frequencies and their assignments for Ben, Ben@Q[7], and Ben@Q[7]@MNPs.

| Benzene | Ben@Q[7] | Ben@Q[7]@MNPs | Assignment^[6]^ |
| --- | --- | --- | --- |
| 609 cm^-1^ | 609 | 608 | *ν*₆, ring deformation (e₂g) |
| 993 | 1001 (Δ*ν* = +8) | 993 | *ν*₁, ring breathing (a₁g) |
| 1176 | 1175 | 1176 | *ν*₉, C–H deformation (e₂g) |
| 1587 | 1588 | 1570 (Δ*ν* = -17) | *ν*₈, C–C stretching (e₂g) |
| 1607 | 1607 | 1596 (Δ*ν* = -11) | *ν*₁ + *ν*₆, in Fermi resonance with *ν*₈ |
| 2951 | 2982 | 2951  FWHM broadening | C–H stretch^[7]^ |
| 3063 | 3069 (Δ*ν* = +6) | 3063  FWHM broadening | C–H symmetric stretching (A_1g_ ) |

**Table S6** Raman frequencies and their assignments for Q[7], Ben@Q[7], Q[7]@MNPs and Ben@Q[7]@MNPs.

| Q[7] | Ben@Q[7] | Q[7]@MNPs | Ben@Q[7]@MNPs | Assignment^[8]^ |
| --- | --- | --- | --- | --- |
| 444 | 444 | 447  FWHM broadening | 447  FWHM broadening | *σ* N–C–N |
| 657 | 656 | 667 (Δ*ν* = +10) | 667(+10) | *τ* HC–CH |
| 833 | 830  (Δ*ν* = -3) | 833 | 835 | *δ* C–N–C + *ρ* CH₂ |
| 900 | 899 | 906 (Δ*ν* = +6) | 906 (Δ*ν* = +6) | *β* C–N–C + *τ* N–C–C–N + *ν* C–C |
| 1383 | 1380  (Δ*ν* = -3) | 1380 (Δ*ν* = -3) | 1380 | Symmetric  *ν* C–N |
| 1422 | 1419  (Δ*ν* = -3) | 1419 (Δ*ν* = -3) | 1420 (Δ*ν* = -2) | Asymmetric  *ν* C–N |
| ~1745  five-ridged peak | ~1745  two-ridged peak | ~1428  two-ridged peak | ~1428  two-ridged peak | *ν* C=O |

*ν* = stretch, *β* = bend, *δ* = deformation, *ρ* =rock, *δ* = scissor and *τ* = twist.

**6. Cycling performance**

**Fig. S12** Adsorption percentages of Ben and *n*-Hep by Q[7]@MNPs in the first cycle.

**Fig. S13** Adsorption percentages of Ben and *n*-Hep by Q[7]@MNPs in the second cycle.

**Fig. S14** Adsorption percentages of Ben and *n*-Hep by Q[7]@MNPs in the third cycle.

**Fig. S15** Adsorption percentages of Ben and *n*-Hep by Q[7]@MNPs in the fourth cycle.

**Fig. S16** Adsorption percentages of Ben and *n*-Hep by Q[7]@MNPs in the fifth cycle.

**7. Purification of *n*-Hep**

**Fig. S17** Gas chromatography analysis before Q[7]@MNPs adsorption of *n*-Hep/Ben mixture (v:v = 98:2).

**Fig. S18** Gas chromatography was used to determine the relative absorption of the solution after Q[7]@MNPs adsorbed *n*-Hep/Ben(v:v = 98:2).

**8.** **References**

[1]	G. Kresse, J. Hafner, Ab Initio molecular Dynamics for Liquid Metals. *Phys. Rev. B*. **47**, 558–561(1993).

[2]	G. Kresse, J. Hafner, Ab initio molecular-dynamics simulation of the liquid-metalamorphous-semiconductor transition in germanium. *Phys. Rev. B.* **49**, 14251–14269(1994).

[3]	J. P. Perdew, K. Burke, M. Ernzerhof, Generalized Gradient Approximation Made Simple. *Phys. Rev. Lett.* **77**, 3865–3868(1996).

[4]	G. Kresse, D. Joubert, From ultrasoft pseudopotentials to the projector augmentedwave method. *Phys. Rev. B.* **59**, 1758–1775(1999).

[5]	S. Grimme, J. Antony, S. Ehrlich, H.A. Krieg, Consistent and accurate ab initio parametrization of density functional dispersion correction (DFT-D) for the 94 elements H-Pu. *J. Chem. Phys.* **132**, 154104(2010).

[6]	C. Zhang, P. M. Allotta, G. Xiong, P. C. Stair, UV‑Raman and Fluorescence Spectroscopy of Benzene Adsorbed Inside Zeolite Pores. *J. Phys. Chem. C* **112**, 14501–14507(2008).

[7]	H. J. Meyer, R. Brun Jr, A. L. Zhang, R. R. Alfano, Raman investigation of vibrational energy interactions of liquids in nanoscale confinement. *J. Phys. Photonics* **2**, 045002(2020).

[8]	S. Mahajan, T.-C. Lee, F. Biedermann, J. T. Hugall, J. J. Baumberg, O. A. Scherman, Raman and SERS spectroscopy of cucurbit[*n*]urils. *Phys. Chem. Chem. Phys.* **12**, 10429–10433(2010)
